# Virtual Screening of Drug-Like Compounds as Potential Inhibitors of the Dengue Virus NS5 Protein

**DOI:** 10.3389/fchem.2022.637266

**Published:** 2022-02-10

**Authors:** Leidy L. García-Ariza, Cristian Rocha-Roa, Leonardo Padilla-Sanabria, Jhon C. Castaño-Osorio

**Affiliations:** ^1^ Grupo de Inmunología Molecular, Centro de Investigaciones Biomédicas, Universidad del Quindío, Armenia, Colombia; ^2^ Grupo de Parasitología Molecular, Centro de Investigaciones Biomédicas, Universidad del Quindío, Armenia, Colombia; ^3^ Biophysics of Tropical Diseases, Max Planck Tandem Group, Universidad de Antioquia, Medellín, Colombia

**Keywords:** dengue virus, NS5 protein, drug-like compounds, molecular docking, virtual screening

## Abstract

Dengue virus (DENV) is the causative agent of dengue fever. Annually, there are about 400 million new cases of dengue worldwide, and so far there is no specific treatment against this disease. The NS5 protein is the largest and most conserved viral protein among flaviviruses and is considered a therapeutic target of great interest. This study aims to search drug-like compounds for possible inhibitors of the NS5 protein in the four serotypes of DENV. Using a virtual screening from a ∼642,759-compound database, we suggest 18 compounds with NS5 binding and highlight the best compound per region, in the methyltransferase and RNA-dependent RNA polymerase domains. These compounds interact mainly with the amino acids of the catalytic sites and/or are involved in processes of protein activity. The identified compounds presented physicochemical and pharmacological properties of interest for their use as possible drugs; furthermore, we found that some of these compounds do not affect cell viability in Huh-7; therefore, we suggest evaluating these compounds *in vitro* as candidates in future research.

## Introduction

Dengue virus (DENV) is a member of the genus *Flavivirus* belonging to the family *Flaviviridae* (Zou et al., 2011; Lim et al., 2013a). DENV is the causative agent of the viral disease known as dengue fever, which is transmitted through the bite of mosquito species *Aedes aegypti* and *Aedes albopictus* (Regato et al., 2008; Basavannacharya and Vasudevan., 2014). This disease mainly affects people who live in tropical and subtropical countries, with approximately 400 million new cases worldwide annually, and can lead to febrile illness and flu-like symptoms or can progress to the more severe dengue hemorrhagic fever or dengue shock syndrome (Klema et al., 2016). However, to date, there is no specific treatment that can inhibit the replication of DENV.

This virus has a non-segmented, single-stranded, positive-sense RNA genome of approximately 11 kb (Basavannacharya and Vasudevan., 2014), which codes for 10 proteins: 3 structural virion components (C, PRM, and E proteins) and 7 nonstructural proteins (NS1, NS2A, NS2B, NS3, NS4A, NS4B, and NS5) (Lim et al., 2013b; Basavannacharya and Vasudevan., 2014; Galiano et al., 2016). To date, four serotypes of DENV have been reported (DENV1 to DENV4) (Niyomrattanakit et al., 2015; Lai et al., 2017; Potisopon et al., 2017). Nevertheless, in the last years, the presence of a fifth serotype with a sylvatic cycle was reported (Mustafa et al., 2014).

The NS5 protein is the largest (Troost and Smit, 2020) and most highly conserved viral protein encoded by the flavivirus genome (Zou et al., 2011; Bollati et al., 2010; García et al., 2017; Wu, J et al., 2020; Bhatnagar et al., 2021). In particular, this protein shows approximately 67–82% amino acid sequence identity among the four dengue serotypes. NS5 consists of two domains, namely methyltransferase (MTase) domain and RNA-dependent RNA polymerase (RdRp) domain (Klema et al., 2016). These domains are linked through a short sequence of poorly conserved amino acids (Lim et al., 2015). The MTase and RdRp domains have enzymatic activity, and both are essential for the viral replication cycle (Bhattacharya et al., 2008).

The N-terminal region of the protein comprises the MTase domain (with a length of approximately 270 amino acids) (Galiano et al., 2016), which functions as a double methyltransferase that can methylate the 5′-end of the viral RNA genome at the N-7 position of the guanosine cap (N-7 MTase) as well as the 2′-OH position of the ribose of the first nucleotide (2′O MTase) (Lim et al., 2013b). Depending on the serotype and the experimental system, the MTase domain, within the context of NS5, can positively influence the polymerase activity. Specifically, the MTase domain of DENV2 can stimulate RNA loading within the adjacent polymerase domain and improve its stable catalytic state during *de novo* specific initiation and the elongation reaction (Potisopon et al., 2017).

On the other hand, the C-terminal region of NS5 harbors RdRp (with a length of approximately 630 amino acids) (Galiano et al., 2016), which plays a vital role in the viral life cycle through replication. After viral entry and the translation of proteins from its genome, the polymerase domain performs the *de novo* synthesis of RNA (first generating RNA of negative polarity from RNA of positive polarity (Lim et al., 2015). Like all polymerases, the structure of the RdRp of flaviviruses resembles a right hand with the characteristic subdomain fingers (amino acids 273- 315, 416-496, and 543- 600), palm (amino acids 497-542 and 601-705), and thumb (amino acid 706-900) (Zou et al., 2011; Najera, 2013; Galiano et al., 2016). In addition, the RdRp domain is unique to RNA viruses, and it is absent in human cells; for this reason, the DENV NS5 protein is an attractive target in the search for antiviral compounds (Malet et al., 2008; De Burghgraeve et al., 2013; Meguellati et al., 2014; Alhossary et al., 2018; Mirza et al., 2019). The crystal structure of the RdRp catalytic domain of DENV was reported by Yap *et al.*, allowing the exploration of regions in its structure that could be of interest for the design of anti-dengue compounds (Yap et al., 2007).

To date, several inhibitors of MTase and RdRp activities have been identified by large-scale *in vitro* screening (Bhatnagar et al., 2021). For instance, sinefungin (a SAM analog with a broad antiviral spectrum) has shown affinity six times greater than SAM for its binding site in the MTase domain (Lim et al., 2015). Ribavirin, a synthetic analog of guanosine, has been shown to inhibit dengue and hepatitis C virus replication (Chang et al., 2011; Tomlinson and Watowich, 2011); however, the use of ribavirin is limited by its oral toxicity, and its aerosol presentation diminishes its efficacy for clinical uses (Fusco and Chung, 2014).

Also, the activity of the RdRp enzyme of DENV is inhibited allosterically by blocking the RNA tunnel using N-sulfonylanthranilic acid derivatives, which are considered desirable for the development of antiviral compounds (Yin et al., 2009b; Niyomrattanakit et al., 2010). Likewise, the activity of this enzyme is inhibited by the action of beta-d-2′-ethenyl-7-deaza-adenosine triphosphate (2′E-7D-ATP) through competition with the natural nucleotide. This nucleoside analog, initially developed for hepatitis C (HCV), showed anti-dengue activity in cell culture and significantly reduced viremia in mouse models with DENV. However, the catalytic efficiency of incorporation of this molecule is 10 times lower than that of ATP (Latour et al., 2010; De Burghgraeve et al., 2013; García et al., 2017). Two non-nucleoside inhibitors, retinamide and ivermectin, were identified in binding assays as compounds that can block DENV NS5 (Lim et al., 2015). Ivermectin is reported as an inhibitor of the α/β importin and therefore of the NS5 polymerase since it is required for its activity. There are reports that a previous treatment with ivermectin inhibits dengue virus infection in Vero cells; in addition, a pretreatment with this compound strongly inhibits the nuclear localization of NS5 during infection with DENV1 and DENV2 in BHK-21 or Huh-7 cells (Fusco and Chung, 2014).

Despite many efforts in the search for antiviral compounds against DENV, success has been limited (Diosa-Toro et al., 2018); consequently, it is necessary to look for new alternatives with low or no toxicity and powerful anti-dengue activity. For this, compounds with similar properties to drugs already approved for use in humans can be of great help. Also, the application of computational tools, such as virtual screening, predictors of physical–chemical characteristics, and molecular dynamics (MD) simulations, can contribute to the design and improvement of new drugs. In recent years, most inhibitors have been first selected *via* in *silico* or high-throughput screening, which was followed by the evaluation of their antiviral activities *via in vitro* or in-cell based assays (Tian et al., 2018). Here, we explore seven binding sites in the NS5 protein of the four DENV serotypes and perform virtual screening strategies to select compounds that can be tested against DENV. Finally, we suggest a short list of 18 compounds that could be considered as candidates for *in vitro* evaluation.

## Materials and Methods

### Structural Modeling of DENV1–4 NS5 Proteins

The structural models of the NS5 proteins of DENV1, DENV2, and DENV4 serotypes were constructed by homology modeling on the SWISS-MODEL web server (Waterhouse et al., 2018), using the full crystal structure of NS5 DENV3 (PDB accession code: 5JJR) (Lim et al., 2016) as a template. We used the consensus amino acid sequences of NS5 for each serotype obtained from the Virus Variation database (NCBI) (Hatcher et al., 2017). For the NS5 protein of DENV3, the non-crystallized regions were modeled but retained the rest of the crystallized structure.

The sequence alignment was performed by the UniProt web server (https://www.uniprot.org/) (Uniprot Consortium, 2021). The stereochemical quality of the constructed models was assessed by analyzing the Ramachandran plot (Hollingsworth and Karplus, 2010), which allows to evaluate the phi (Φ) and psi (Ψ) angles of each amino acid. These plots were obtained from the MolProbity web server (Williams et al., 2018). Additionally, we calculated the Z-scores, obtained from the ProSA web server (Wiederstein and Sippl, 2007). The root mean square deviation (RMSD) between the structures of the serotypes was calculated with the MatchMaker module available in Chimera v1.11.2 software (Pettersen et al., 2004).

### Virtual Screening of Drug-Like Compounds and Molecular Docking Calculations

The virtual screenings were performed using the supercomputer of the Texas Advanced Computing Center (TACC) (https://drugdiscovery.tacc.utexas.edu) (Viswanathan et al., 2014) linked to the ZINC database of compounds for virtual screening (https://zinc.docking.org/). AutoDock Tools v.1.5.6 (Morris et al., 2009) was used for preparing the receptors (NS5 protein) and ligands (compounds used as controls). For proteins, we removed the water molecules, and co-crystallized ligands, polar hydrogens, and Kollman charges were added. For ligands, the polar hydrogens, Gasteiger charges, and rotatable bonds were added. The compounds in the ZINC Lrg database (∼642,759) were not prepared as the TACC portal, where this library is available, has the compounds ready for molecular docking.

The molecular docking calculations were performed with AutoDock Vina software (Trott and Olson, 2010) in several important regions for functionality of NS5 in the four serotypes of DENV. These regions have been studied in other research studies (Zou et al., 2011; Galiano et al., 2016; Lim et al., 2015; Malet et al., 2008; Yap et al., 2007; Niyomrattanakit et al., 2010; Dong et al., 2008; Zhou et al., 2007; Zhao et al., 2015a), and they have been recognized as interesting sites for antiviral drug development. For the RdRp domain, we evaluated cavities A and B (Malet et al., 2008; Zhou et al., 2007), the RNA tunnel (Galiano et al., 2016; Yap et al., 2007; Niyomrattanakit et al., 2010), and the GDD motif (Galiano et al., 2016). Likewise, we assessed the KDKE tetrad (Dong et al., 2008; Zhou et al., 2007; Zhao et al., 2015a; Zhao et al., 2015c) and the SAM- and GTP-binding site (Lim et al., 2015; Dong et al., 2008) for the MTase domain. Overall, we explored seven regions in the two domains. The regions with a crystallized ligand, such as GTP-binding site, SAM-binding site, and GDD motif, were validated using re-docking. All the models were aligned to be able to use the same boxes in the four serotypes, and the boxes were configured with a dimension of 24 Å. The compounds were obtained from the library ZINC Lrg, available as a tab inside TACC options (https://drugdiscovery.tacc.utexas.edu). This library contains ∼642,759 commercially available drug-like compounds. The visualizations of the 2D interactions and the generation of the protein–ligand complexes were performed with Chimera v1.11.2 software (Pettersen et al., 2004).

### Selection of the Compounds

**FIGURE 1:**
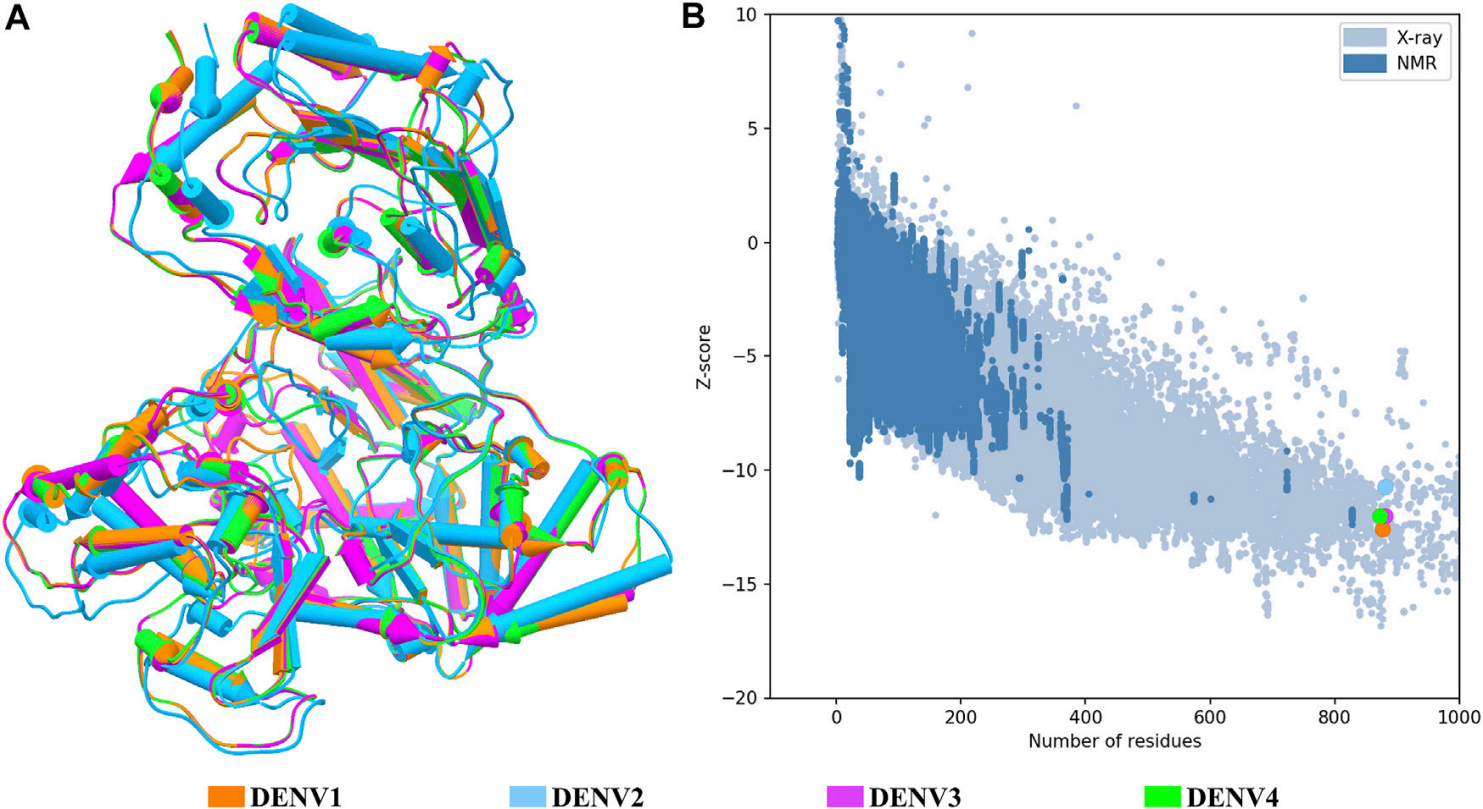
Structural quality of the NS5 models of DENV. **(A)** Superposition of the four structures of the DENV NS5 protein. The 3D structure is shown in pipes for alpha-helix and planks for the beta-sheet. **(B)** Graphic generated by the ProSA web server; each model showed a Z-score value as follows: DENV1 of –12.6, DENV2 of –10.68, DENV3 of –12.09, and DENV4 of –11.94. The dots in B follow the same color code as shown in A.

Initially, we selected the candidate compounds based on two filters: 1) multi-domain binding compounds (compounds that were docked to both the MTase and RdRp domains) and 2) single-domain binding compounds or those that were docked only to one domain. These analyses were performed using a classification script in the R package (R Foundation for Statistical Computing, s.f.). We applied a strict parameter to select compounds docked to the same region of the NS5 protein in the four DENV serotypes, which should theoretically increase their spectrum of activity and antiviral potential. The selected compounds were also subjected to other filters, including compliance to Lipinski’s rules, solubility, gastrointestinal absorption, and prediction of toxicological risks (Figure 1).

### Compliance to Lipinski’s Rules, Solubility, Gastrointestinal Absorption, and Toxicological Risk Predictions

The prediction of Lipinski’s rules, solubility, gastrointestinal absorption, and toxicological risk was performed using the SwissADME web server (Daina et al., 2017). For compliance with Lipinski’s rules, this tool provides a qualification of *Yes* or *No*, accompanied by the number of rules violated. We discarded all the compounds that presented at least one violation. For solubility prediction, we used three predictors in SwissADME, which yields a qualification of insoluble, poorly, moderately, soluble, very, and highly. We decided to assign a score for each prediction as follows: insoluble and poorly, a value of 1; moderately and soluble, a value of 2; and very and highly, a value of 3. We discarded all the compounds that presented a value ≤5. In order to estimate the gastrointestinal absorption of each query compound, we accepted only compounds with a high predicted gastrointestinal absorption. Finally, we used DataWarrior software (Sander et al., 2015), ProToxII (Banerjee et al., 2018), and CarcinoPred-EL (Zhang et al., 2017) web servers to predict several types of toxicological risks, including possible mutagenic, tumorigenic, reproductive, irritant, hepatotoxic, immunotoxic, cytotoxic, and carcinogenic risks. We discarded the compounds that presented three or more toxicological risks.

### Molecular Dynamics Simulations and Binding Free-Energy Calculations

Molecular dynamics simulations were performed using GROMACS 2019 software (Berendsen et al., 1995). The complexes formed by the four DENV serotypes and the best selected compound per each binding site were taken as initial coordinates and were simulated. A total of 20 simulations were carried out. For the protein, the amber ff99SB-ILDN force field was used (Lindorff-Larsen et al., 2010); the ligands were parameterized using the general AMBER force field (Wang et al., 2004) and ACPYPE web server (based on ANTECHAMBER, https://www.bio2byte.be/acpype/), by which the parameters of the ligands to work in GROMACS are obtained (Sousa da Silva and Vranken, 2012). Each protein–ligand complex was solvated using the TIP3P water model, and its charges were neutralized using Na^+^ Cl^−^ ions, and an excess of ions were added to reach a concentration of 0.15 M of NaCl. Each system was subjected to an energy minimization stage of 50,000 steps, followed by an NVT equilibration for 250 ps using a temperature of 310 K. Then, a series of equilibrations were performed under NPT conditions using a pressure of 1 bar, with decreasing restrictions on the heavy atoms of the protein and the ligand for 250 ps each (1,000, 100, 10, and 1 kJ/mol*nm^2^). In general, all systems were subjected to one energy minimization stage and five equilibration stages. The systems were subjected to a production stage for a total of 40 ns, in which a V-rescale thermostat and a Parrinello–Rahman barostat were implemented. A time-step of 2 fs was used. Once the simulations were finished, the RMSD, root mean square fluctuation (RMSF), radius of gyration (Rg), and contacts plots of each protein–ligand complex were obtained through the gmx_rms, gmx_rmsf, gmx_gyrate, and gmx_mindist modules contained in the GROMACS package, respectively. Then, we calculated the binding free-energy (ΔG_bind_) using the molecular mechanics/Poisson–Boltzmann surface area (MM/PBSA) method, available in the tool gmx_MMPBSA (Miller et al., 2012; Valdés et al., 2021). The ΔG_bind_ between a protein and ligand can be calculated as seen in the following equations (Hou et al., 2011):

$$\Delta G_{\mathrm{bind}}=\Delta \mathrm{H-T}\Delta S\approx \Delta E_{\mathrm{MM}}+\Delta G_{\mathrm{solvation}}-T\Delta \mathrm{S;}$$ (1)

$$\Delta E_{\mathrm{MM}}=\Delta E_{\mathrm{internal}}+\Delta E_{\mathrm{electrostatic}}+\Delta E_{\mathrm{van}\;\mathrm{der}\;\mathrm{Waals}};$$ (2)

$$\Delta G_{\mathrm{Solvation}}=\Delta G_{\mathrm{PB/GB}}+\Delta G_{\mathrm{SA}},$$ (3)

where $\Delta E_{\mathrm{MM}}$ represents the contribution of MM energy, and it can be obtained from the force field implemented in the MD simulations. $\Delta G_{\mathrm{Solvation}}$ represents the solvation energy given by the sum of the polar contribution ( $\Delta G_{\mathrm{PB/GB}}$ ) and nonpolar contribution ( $\Delta G_{\mathrm{SA}}$ ). $\Delta G_{\mathrm{PB/GB}}$ can be obtained using the Poisson–Boltzmann or generalized-Born models, while $\Delta G_{\mathrm{SA}}$ can be calculated by the solvent accessible surface area (SASA) (Hou et al., 2011; Genheden and Ryde., 2015). The term TΔS can be added to refine the predictions. For $\Delta G_{\mathrm{bind}}$ calculations, frames from the 10 ns to the end were taken for each 30 ps, for a total of 1,000 frames per each trajectory.

### Pharmacophore Modeling

Once the list of the best compounds that passed all the previous filters was obtained, we used the binding poses on the four serotypes of the compounds with the best binding-free energy scores, and we generated a model of the pharmacophore resultant of a possible inhibitor for each binding site separately. For that, we use the PharmaGist web server (http://bioinfo3d.cs.tau.ac.il/PharmaGist/) (Schneidman-Duhovny et al., 2008). This allows a rational design of molecules, by identifying modifications that can improve the affinity of each compound for its binding site.

### Cytotoxicity Assay in Huh-7 Cells

The cytotoxic effect of acquired compounds was evaluated on the Huh-7 cell line. A total of 2 × 10^4^ cells were subcultured per well in 96-well plates and incubated for 24 h at 37°C and 5% CO₂. Once the cells reached 80% confluence, each of the compounds was added in serial concentrations from 0.7 to 50 µM and incubated again under the previously described conditions for 24 h. The medium was then replaced, and 15 μL of the staining solution with tetrazolium salt (MTT) was added (CellTiter 96^®^ Non-Radioactive Cell Proliferation Assay kit, Reference G4001, Promega) and incubated again for 4 h under the previously mentioned conditions. After incubation, 100 µL of solubilization solution was added and incubated again for an additional 1 h. The contents of the wells were mixed until obtaining uniformity in the coloration, and finally the absorbance of each well was read at 570 nm. As a control in the test, 0.3% dimethylsulfoxide (DMSO) was used, which corresponds to the vehicle control (solution in which the compounds are dissolved at a concentration of 50 μM), and the cellular control was included, which corresponds to untreated Huh-7 cells. For data analysis, the absorbance of the medium was subtracted, and the percentage of cell viability was calculated by applying the following formula:

% Cell Viability = Sample Abs/Control Abs × 100,

where

-Sample Abs corresponds to the absorbance value of each well with cells and treatment.
-Control Abs corresponds to the value of the absorbance of the wells with cells without treatment.

The data were evaluated by the Kruskal–Wallis test, with a comparison by Dunn’s test. All data were analyzed in GraphPad Prism 6.0 software. A *p* value <0.05 was considered statistically significant.

## Results

### Structural Modeling of DENV1–4 NS5 Proteins

The structural models of the NS5 proteins of DENV serotypes 1 and 4 were obtained by homology modeling. The models were generated from consensus sequences obtained with BioEdit using the sequences reported in the Virus Variation database (Hatcher et al., 2017) for each serotype. We found 2097 sequences of DENV1, 1559 of DENV2, and 370 of DENV4. The NS5 of DENV3 showed a high percentage of structural and sequence identity with the other serotypes of DENV as can be seen in Table 1. The sequence alignment is shown in Supplementary Figure S1, in which some relevant regions are highlighted.

**TABLE 1:** Percentages of sequence identity and structural RMSD (Å) between the NS5 protein of the four DENV serotypes.

NS5 serotype	DENV1		DENV2		DENV3		DENV4	
	Sequence identity	RMSD	Sequence identity	RMSD	Sequence identity	RMSD	Sequence identity	RMSD
DENV1	100	0	80.07	0.90	81.71	0.15	75.59	0.20
DENV2	80.07	0.90	100	0	79.42	0.91	74.38	0.89
DENV3	81.71	0.15	79.42	0.91	100	0	77.08	0.21
DENV4	75.59	0.20	74.38	0.89	77.08	0.21	100	0

Once all the full-length models were obtained, the general quality was analyzed through the estimation of GMQE (Global Model Quality Estimate) and global QMEANDisCo (Qualitative Model Energy Analysis) provided in the SWISS-MODEL web server (overall measure of model quality between 0 and 1), the stereochemical quality by Ramachandran plot, and the structural quality based on the Z-score value, for each model. Based on the quality estimates, the following GMQE and QMEANDisCo values were found for the NS5 models of each serotype: GMQE 0.83 for DENV1 and DENV2, 0.85 for DENV3, and 0.82 for DENV4, and QMEANDisCo 0.82, 0.81, 0.83, and 0.81 ± 0.05 for DENV1, DENV2, DENV3, and DENV4, respectively. The values obtained are close to 1, which indicates favorable quality for these models. On the other hand, the Ramachandran plots (not shown) for these structures suggested the following percentages for the amino acid residues that are located in the favored regions: 91.6% for DENV1, 96.1% for DENV2, 98.3% for DENV3, and 97.6% for DENV4.

Figure 1A shows the four models after a structural alignment. In general, the models showed low RMSD values between them, which supports the idea of designing compounds with activity on the four dengue serotypes. Additionally, Figure 1B shows the spectrum provided by the ProSA web server that locates all the Z-scores for the structures that have been resolved by X-ray and NMR and shows the obtained values for the four models of the NS5 protein of DENV.

### Virtual Screening and Compounds Selection

In this study, we used the DrugDiscovery@TACC web portal of the Texas Advanced Computing Center (https://portal.tacc.utexas.edu) and screened the largest library (Lgr), which contained approximately 642,759 compounds with structural characteristics similar to drugs. The regions with a crystallized ligand, such as GTP-binding site, SAM-binding site, and GDD motif, were validated using re-docking (Supplementary Figure S2). The similarity between these structures was evidenced, with a RMSD value between the experimental and the crystallographic pose of 1.545 Å, 1.707 Å, and 0.884 Å for GTP-binding site, SAM-binding site, and GDD motif, respectively.

**FIGURE 2:**
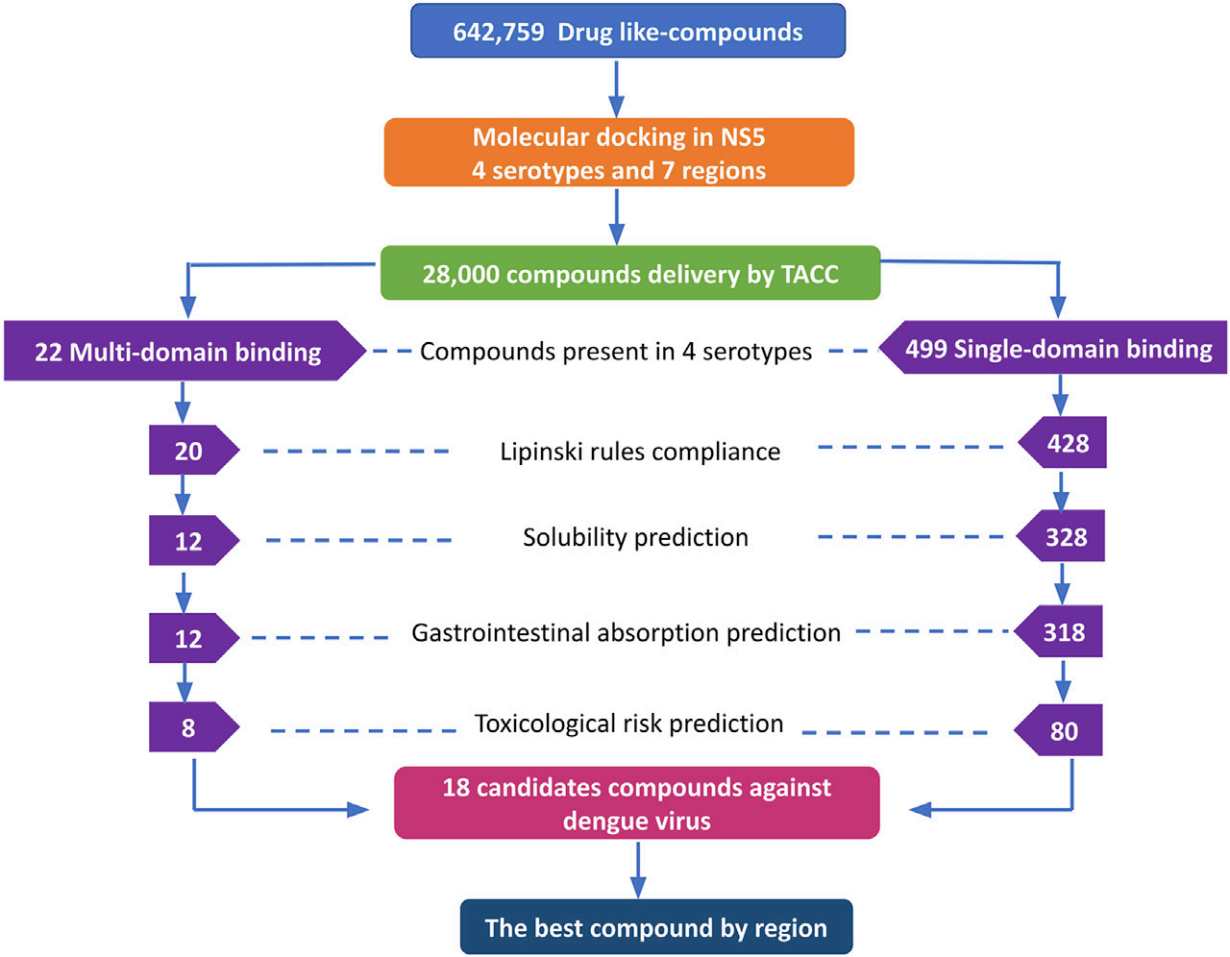
Workflow of the virtual screening of drug-like compounds with possible multi-domain and single-domain binding, as potential candidates for *in vitro* experimental assays against the NS5 protein of DENV. Candidate compounds against the NS5 protein of DENV.

The DrugDiscovery@TACC web portal provided a list of the first 1,000 compounds for each virtual screening run, ranked by their binding-free energy score calculated with AutoDock Vina software. In total, we studied seven regions in the NS5 protein (3 in the MTase domain and 4 in the RdRp domain) of the four DENV serotypes, retrieving a total of 28,000 compounds that were filtered according to our selection criteria. A simple R script was designed to perform the first filter that consisted in: 1) selecting all the compounds that were present in the four serotypes and docked to a region of the MTase domain as well as to a region of the RdRp domain and 2) selecting all the compounds that were present in all four DENV serotypes, but this time, only in one domain of the NS5 protein (MTase or RdRp). This selection resulted in 22 compounds with possible multi-domain binding and 499 compounds with possible single-domain binding in the NS5 protein of DENV (Figure 2).

Once we obtained the compounds for each strategy, we performed each of our filters. For prediction of compliance or violation of Lipinski’s rules, resulted in 20 compounds for strategy 1 and 428 compounds for strategy 2. After evaluating our scores for solubility prediction, strategy 1 was reduced to 12 compounds and strategy 2 to 328 compounds. All the compounds of strategy 1 presented high gastrointestinal absorption, which did not reduce the list, while for strategy 2 the list was reduced to 318 compounds. Toxicological risks prediction resulted in the removal of 4 compounds from strategy 1 according to our exclusion criteria, resulting in a list of 8 compounds. Meanwhile, strategy 2 was reduced to 80 compounds that did not present any predicted toxicological risks. Finally, based on the binding score from molecular docking, we postulate the best compounds from both strategies, as potential candidates to be inhibitors of DENV NS5 protein *in vitro* assays, resulting 8 compounds with possible multi-domain binding (strategy 1) and the top 10 compounds with possible single-domain binding (strategy 2).

The 8 compounds resulting from strategy 1 (multi-domain) showed mixed binding between four regions of the NS5 protein of the four DENV serotypes, such as the GTP-binding site, cavity B, KDKE tetrad, and GDD motif. In particular, two regions were located in the MTase domain (KDKE and GTP) and two in the RdRp domain (CB and GDD). For strategy 2 (single-domain), we obtained 80 compounds that passed the filters of the structural predictions. These compounds showed dockings in regions such as the GTP-binding site, cavity B, KDKE tetrad, GDD motif, and SAM-binding site. The single-domain criteria allowed obtaining a much larger list than the list obtained with strategy 1. However, we only took the best 10 compounds (2 per region) for the subsequent analyses. Accordingly, we postulate a short list of 18 compounds, 8 with double binding sites and 10 compounds with single binding sites in the NS5 protein of the four DENV serotypes. The best compounds for the GTP-binding site are shown in Table 2, with *1md* (ID ZINC15827835) being the compound with the best affinity results.

**TABLE 2:** Best compounds obtained for the GTP-binding site of the four DENV serotypes. The best compound is highlighted in bold.

Compound	Molecular structure	Docking score (kcal/mol)			
		DENV1	DENV2	DENV3	DENV4
**1md ZINC15827835**	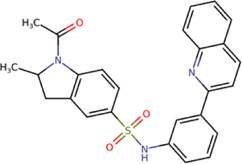	**−9.3**	**−9.3**	**−9.5**	**−8.9**
2md ZINC15827831	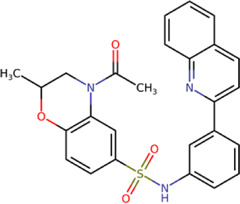	−9.1	−9.1	−9.5	−8.7
3md ZINC15730200	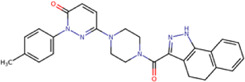	−9.0	−8.8	−8.9	−8.8
4md	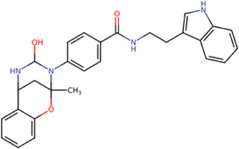	−8.8	−8.6	−9.1	−8.8
ZINC9835726					
5md ZINC9268549	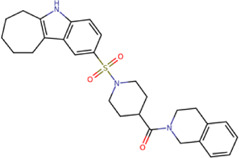	−9.1	−8.8	−9.2	−9.3
1sd ZINC12122852	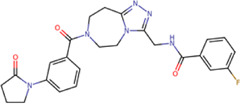	−9.2	−9.2	−9.4	−9.0
2sd ZINC11971657	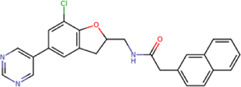	−9.0	−9.0	−9.2	−9.0
GTP^a^	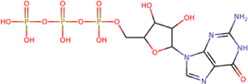	−7.9	−7.7	−7.9	−7.8

a Crystal control: natural subtract of the MTase domain crystallized with the PDB accession code: 4V0R (Zhao et al., 2015a).

### Molecular Dynamics Simulations and Binding-Free Energy Calculations

**FIGURE 3:**
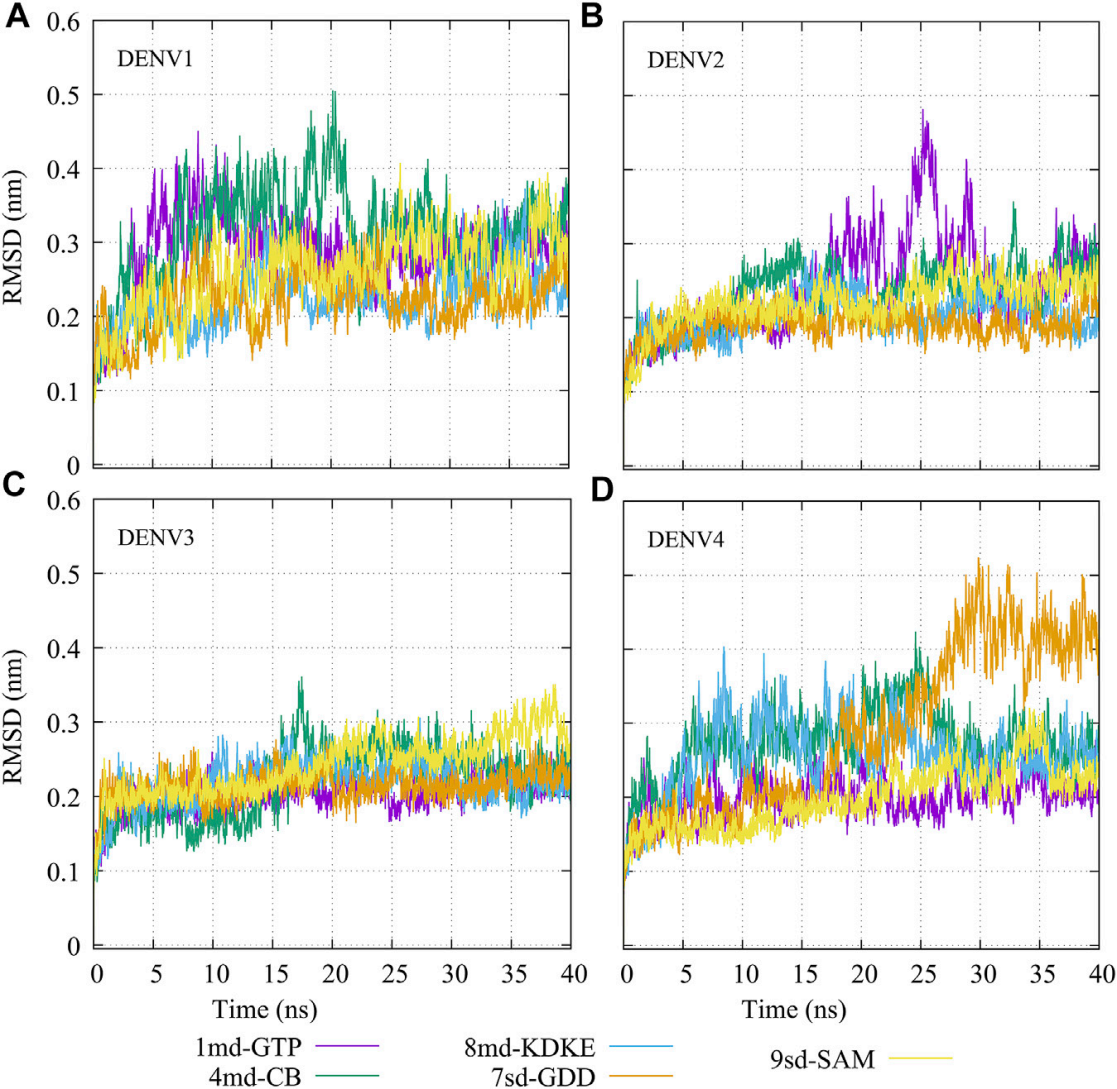
RMSD as a function of the time for the protein–ligand complexes formed by the best compound of each binding site. **(A)** DENV1, **(B)** DENV2, **(C)** DENV3, and **(D)** DENV4.

The molecular dynamics simulations aimed to sample the protein–ligand complexes formed by the best compound at each binding site for 40 ns. Then, with these obtained trajectories and by means of the MM/PBSA method, we calculated a new binding-free energy, which in principle is more computationally robust than the score obtained in the molecular docking. This was in order to analyze on which serotype the interaction of each compound would be stronger. In addition, we obtained the RMSD, RMSF, Rg, and contact map graphs for each simulated complex. In Figure 3, the RMSD values for the NS5 protein of the four DENV serotypes are shown. In brief, the RMSD measures the structural deviation of the protein along the time regarding its initial conformation. For complexes with DENV1 (Figure 3A), an approximate RMSD range of 0.2–0.4 nm was obtained. Compound *4md* (bound in cavity B) presented greater fluctuations at the beginning of the simulation than the other compounds, reaching values very close to 0.5 nm. For the complexes with DENV2 (Figure 3B), the approximate range of RMSD was smaller than that of the DENV1 complexes, being between 0.15 and 0.3 nm. Compound *1md* (bound at the GTP-binding site) shows greater fluctuations in the interval from 17 to 30 ns, reaching values less than 0.5 nm, for the rest of the time, its behavior was similar to the other DENV2 complexes. For complexes with DENV3 (Figure 3C), the approximate range of RMSD was narrower, being between 0.2 and 0.3 nm. For the complexes with DENV4 (Figure 3D), the approximate range of RMSD was 0.2–0.5 nm. All complexes except the one formed by *7sd* (bound in the GDD motif region) exhibited a plateau between 0.3 and 0.4 nm after ∼27 ns. In general, the structural changes for all serotypes were less than or equal to 0.5 nm.

**FIGURE 4:**
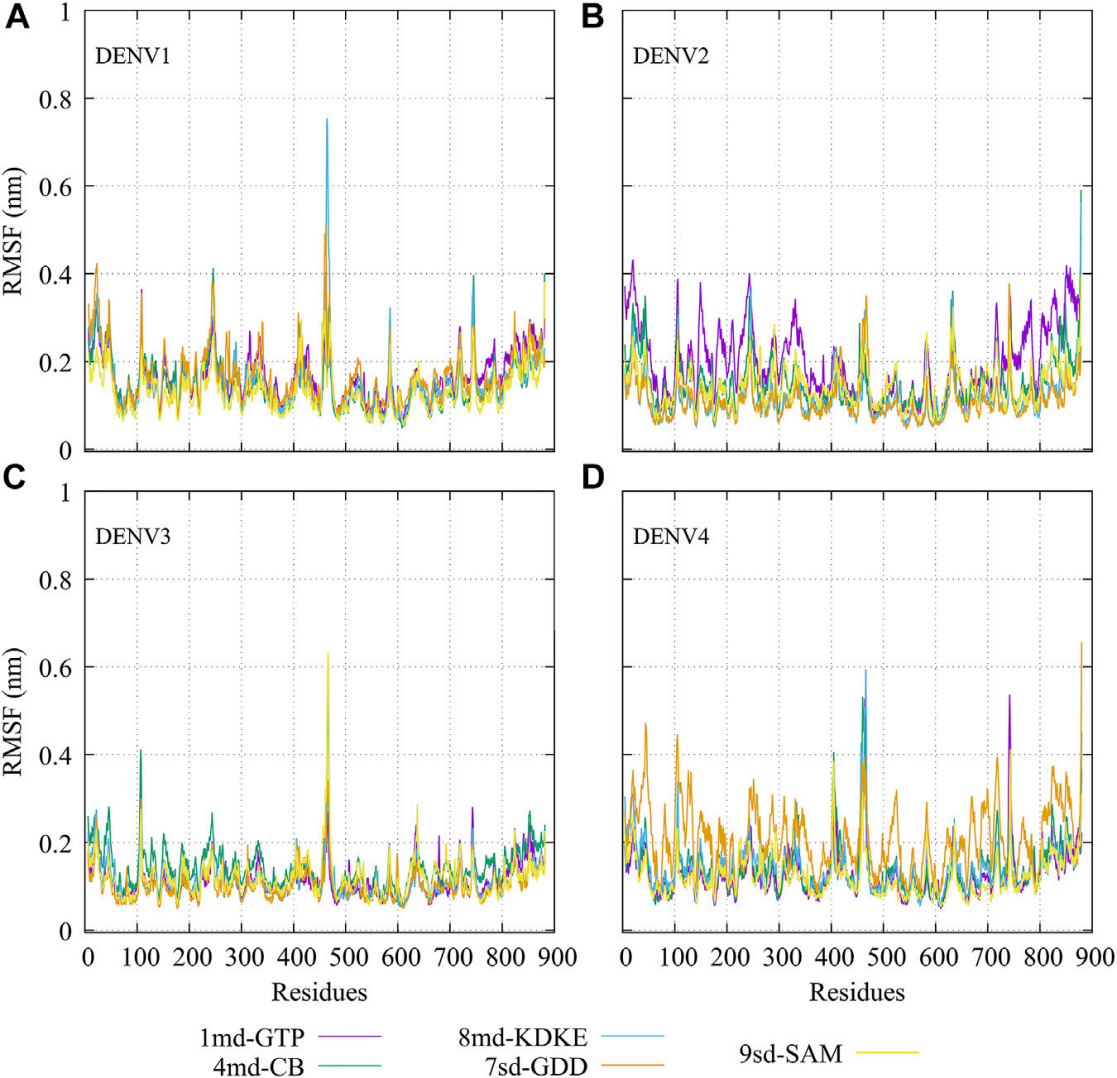
RMSF as a function of numbers of residues for the protein–ligand complexes formed by the best compound of each binding site in **(A)** DENV1, **(B)** DENV2, **(C)** DENV3, and **(D)** DENV4.

The RMSF plots for simulated protein–ligand complexes are shown in Figure 4. In brief, the RMSF measures the average deviation of the protein residues, that is, it measures the fluctuation in their position. Thus, high values in the RMSF indicate portions in the protein with greater flexibility (Martínez, 2015). For the RMSF of the complexes with DENV1 (Figure 4A), it was found that *8md* increased the flexibility of a short fragment (from residue 461–465) reaching a value of ∼0.75 nm. For the complexes formed by DENV2 (Figure 4B), it was observed that *1md* caused a slight increase in the flexibility of the protein with respect to the flexibility of the other complexes, in fragments that go from the N-terminal to residue ∼400 and from residue ∼700 to the C-terminal. For the complexes formed by DENV3 (Figure 4C), compounds *8md* and *9sd* increased the flexibility of the protein in a short fragment from residue 462 to 466, reaching values close to ∼0.63 nm. For the complexes formed by DENV4 (Figure 4D), *7sd* presented an increase in flexibility in the protein with respect to the other complexes.

In order to dynamically track the interactions between the protein and the ligand, we calculated the average frequencies of interaction. Here, the interactions were all those contacts between the protein and the ligand at a distance less than or equal to 0.35 nm; in this way, only the strongest or closest interactions are considered. To do it dynamically, we divide the simulation time into blocks of 10 ns, obtaining 3 blocks of time (since the first 10 ns was omitted for equilibration). If a residue has an interaction percentage of 100% with the ligand, the green color will be assigned, which means that this interaction was conserved for all 10 ns of the time block.

**FIGURE 5:**
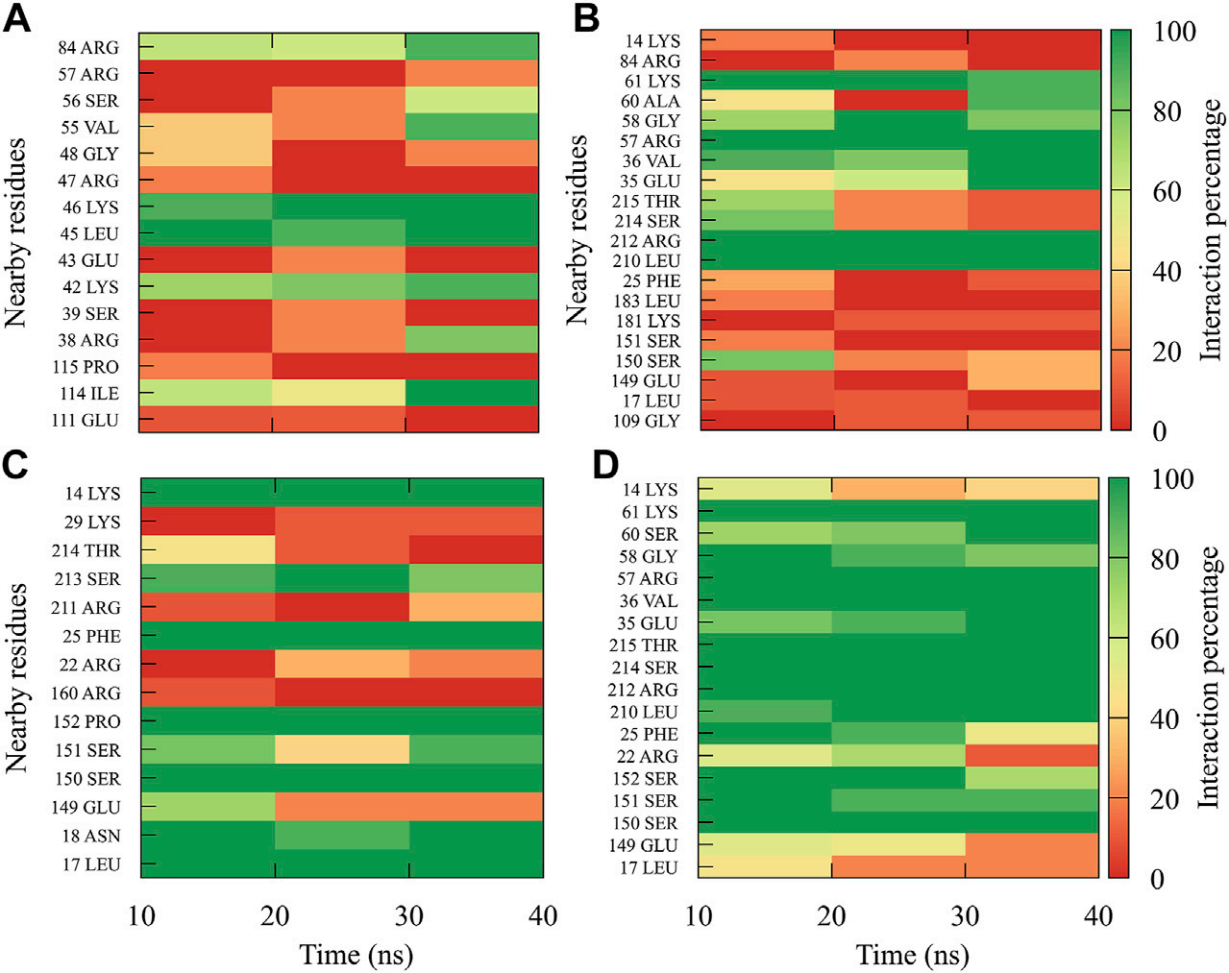
Contact frequency maps for the compound *1md* **,** bound to the GTP-binding site of the **(A)** DENV1, **(B)** DENV2, **(C)** DENV3, and **(D)** DENV4. Red represents that the contact frequency was 0%, while green represents that the contact frequency was 100%.

In general, it can be observed that the interactions between compound *1md* and the residues of the GTP-binding site of the four DENV serotypes (Figure 5) were both in a higher percentage and in greater quantity with DENV4 and DENV3 serotypes, and they were both in a lower percentage and in a lower quantity in DENV2 and DENV1 serotypes. *1md* conserved interactions with residues such as Lys14, Leu17, Asn18, Phe25, Ser151, Leu210 (DENV2 and DENV4), and Ser213, which have been classified as important for the stabilization of the natural substrate GTP (Geiss et al., 2009). To indicate the position of the other amino acids involved, the sequence of NS5 DENV3 was taken as reference, according to the alignment (Supplementary Figure S1).

**FIGURE 6:**
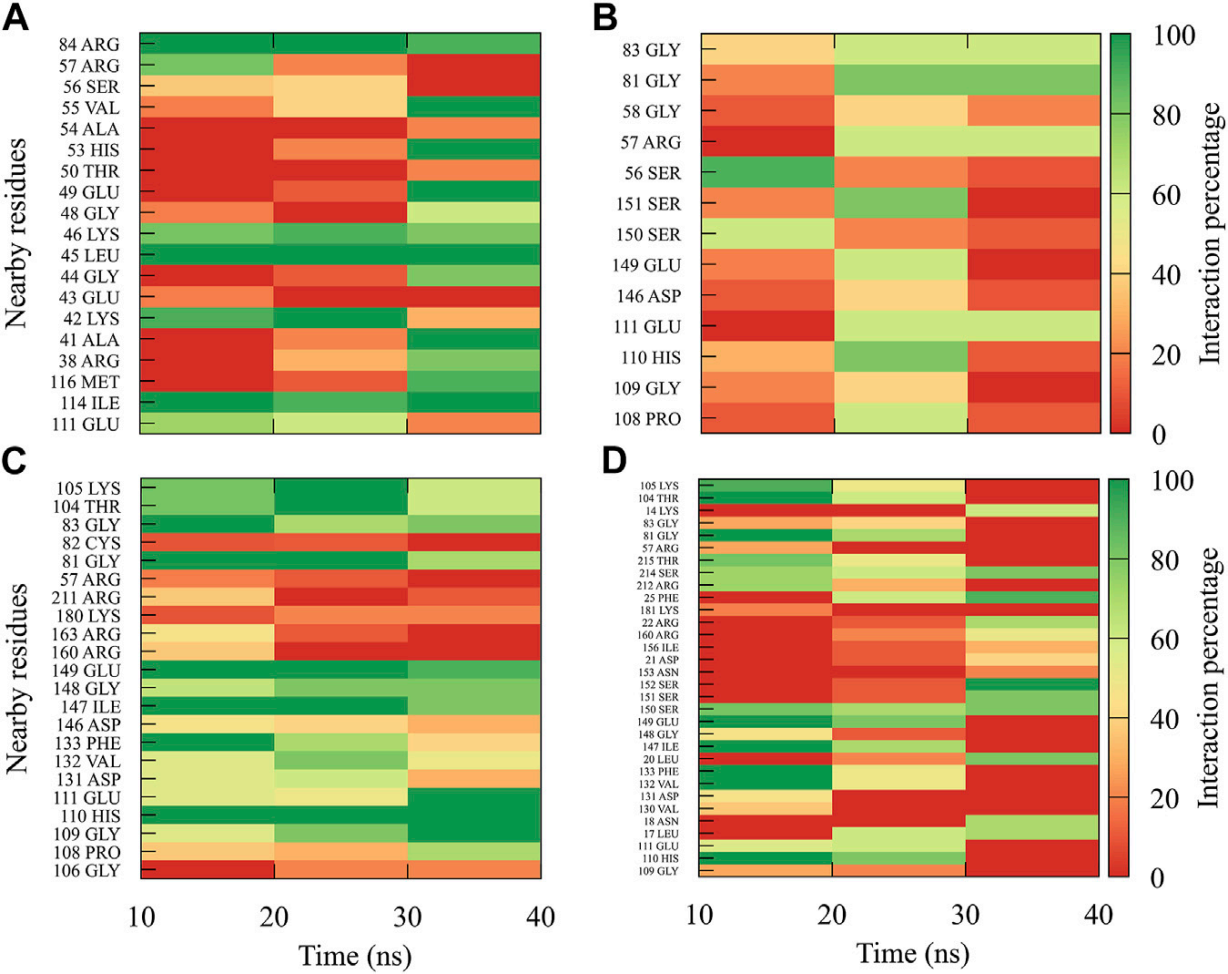
Contact frequency maps for the compound *8md* **,** bound to the region of the KDKE tetrad of the **(A)** DENV1, **(B)** DENV2, **(C)** DENV3, and **(D)** DENV4. Red represents that the contact frequency was 0%, while green represents that the contact frequency was 100%.

The interactions between compound *8md* and the residues in the region of the KDKE tetrad (Figure 6) suggest that the highest number of contacts with the highest frequency occurred in the DENV3 and DENV2 serotypes, while those with the lowest frequencies were DENV1 and DENV4 serotypes. *8md* showed interactions with some amino acids of the KDKE tetrad and close ones, such as Asp146, Ile147, Lys180, and Thr215 (DENV4), which are considered important residues for the interaction of the natural substrate SAM (Zhou et al., 2007; Lim et al., 2011). To indicate the position of the other amino acids involved, the sequence of NS5 DENV3 was taken as reference, according to the alignment (Supplementary Figure S1).

**FIGURE 7:**
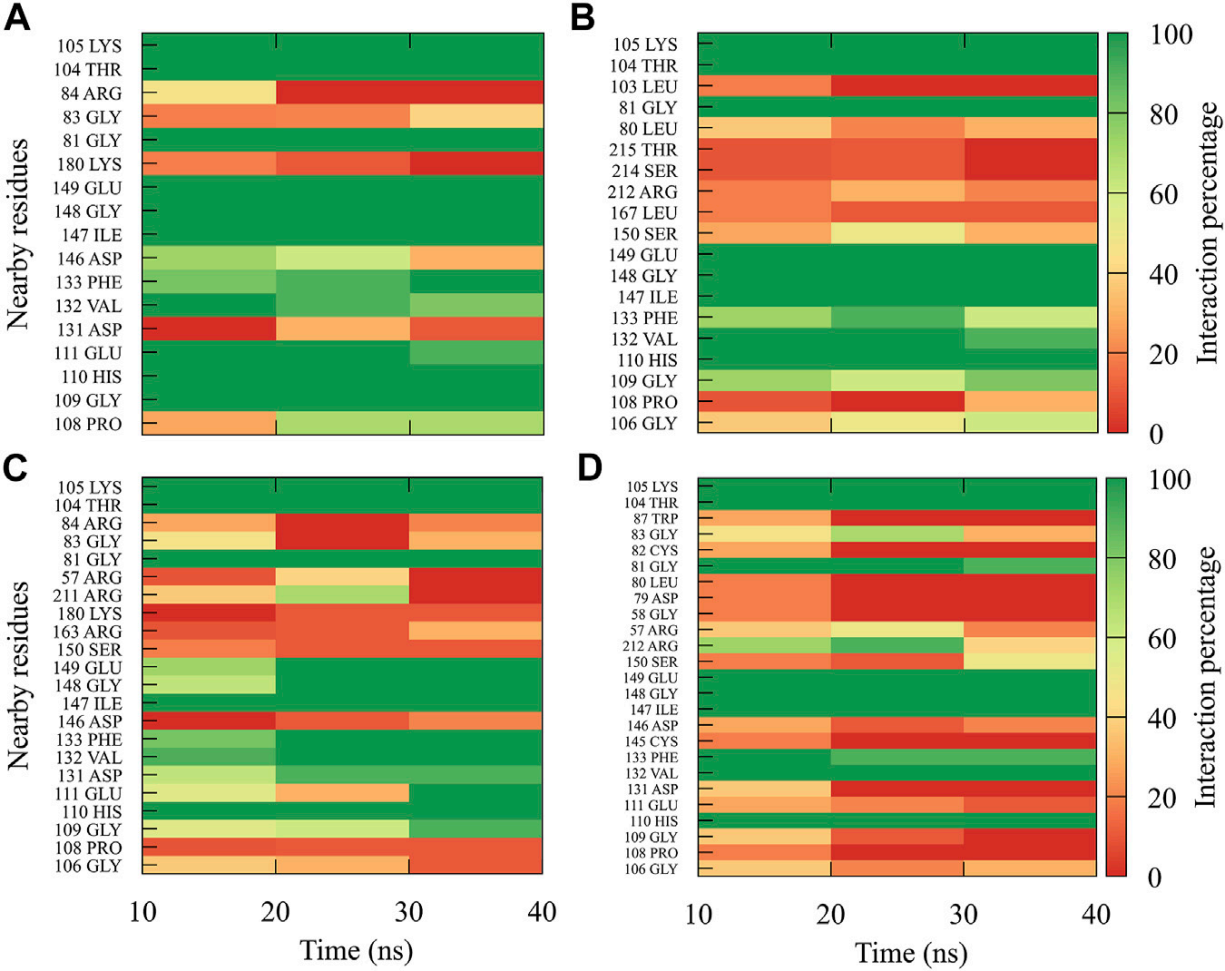
Contact frequency maps for the compound *9sd* **,** bound to the SAM binding-site of the **(A)** DENV1, **(B)** DENV2, **(C)** DENV3, and **(D)** DENV4. Red represents that the contact frequency was 0%, while green represents that the contact frequency was 100%.

For compound *9sd*, bound to the SAM-binding site in the MTase domain, the highest frequencies of interactions were for the DENV1 and DENV2 serotypes (Figures 7A, B); therefore, the lowest frequencies of interactions were for the DENV3 and DENV4 serotypes (Figures 7C, D). Compound *9sd* exhibited interactions with described amino acids important for the stabilization of SAM, such as Lys61, Arg84, Lys105, His110, Glu111, Asp131, Val132, Asp146, Ile147, Gly148, Lys180, and Glu216 (Zhou et al., 2007; Dong et al., 2008; Zhao et al., 2015a; Zhao et al., 2015c). To indicate the position of the amino acids involved, the sequence of NS5 DENV3 was taken as reference, according to the alignment (Supplementary Figure S1).

**FIGURE 8:**
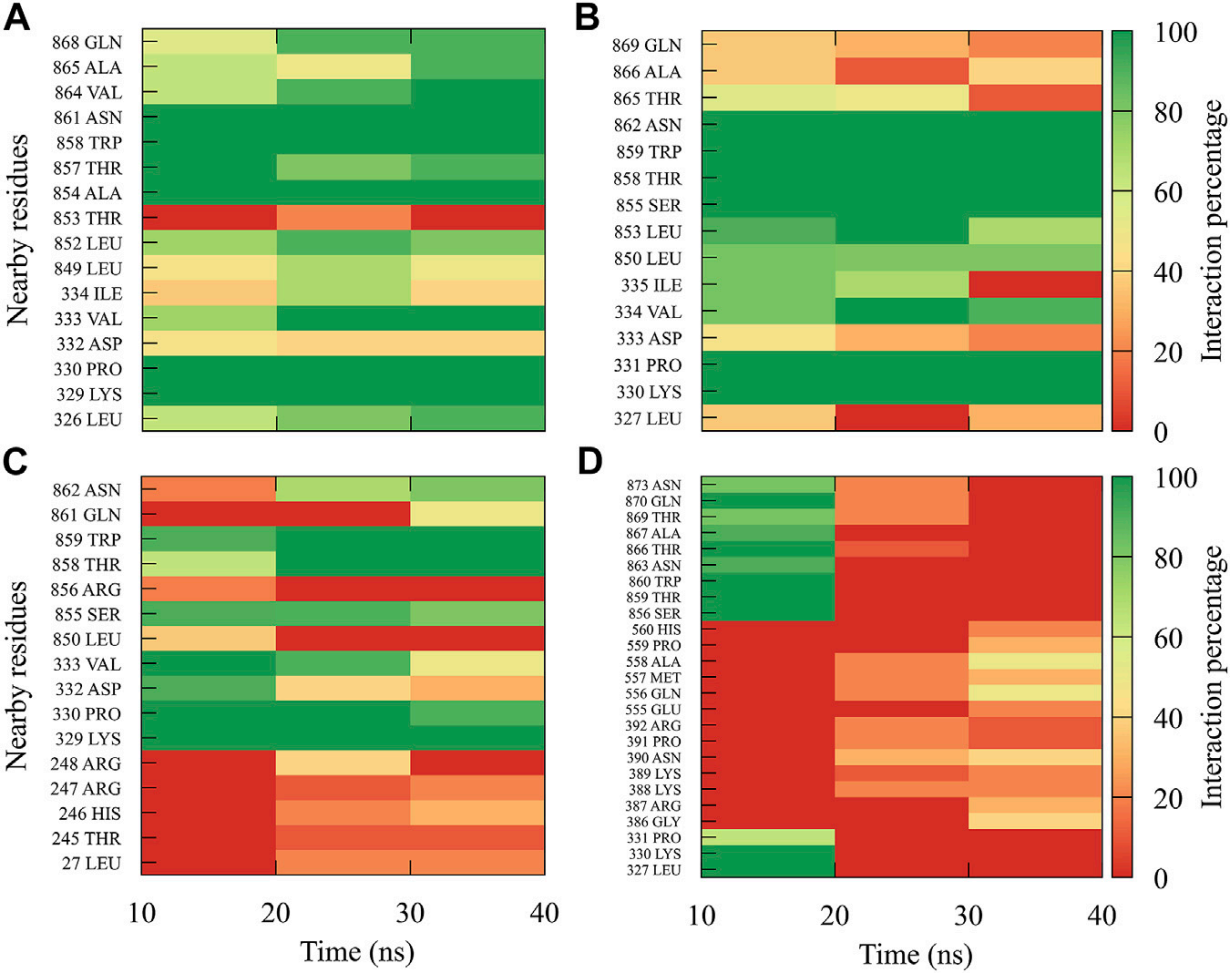
Contact frequency maps for the compound *4md* **,** bound to the cavity B of the **(A)** DENV1, **(B)** DENV2, **(C)** DENV3, and **(D)** DENV4. Red represents that the contact frequency was 0%, while green represents that the contact frequency was 100%.

For compound *4md*, bound to cavity B of the RdRp domain, the highest frequencies and numbers of contacts were for the DENV1 and DENV2 serotypes (Figures 8A, B), while the lowest frequencies and numbers of contacts were for the DENV3 and DENV4 serotypes (Figures 8C, D). *4md* presented interactions with amino acids from cavity B, such as Leu327, Lys329, Pro330, Asp332, Thr858, Trp859, Asn862, Ile863, Ala866, and Gln869, which have been described as interacting with other inhibitors designed for this cavity (Zou et al., 2011; Kaptein et al., 2018; Cannalire et al., 2020). To indicate the position of the amino acids involved, the sequence of NS5 DENV3 was taken as reference, according to the alignment (Supplementary Figure S1).

**FIGURE 9:**
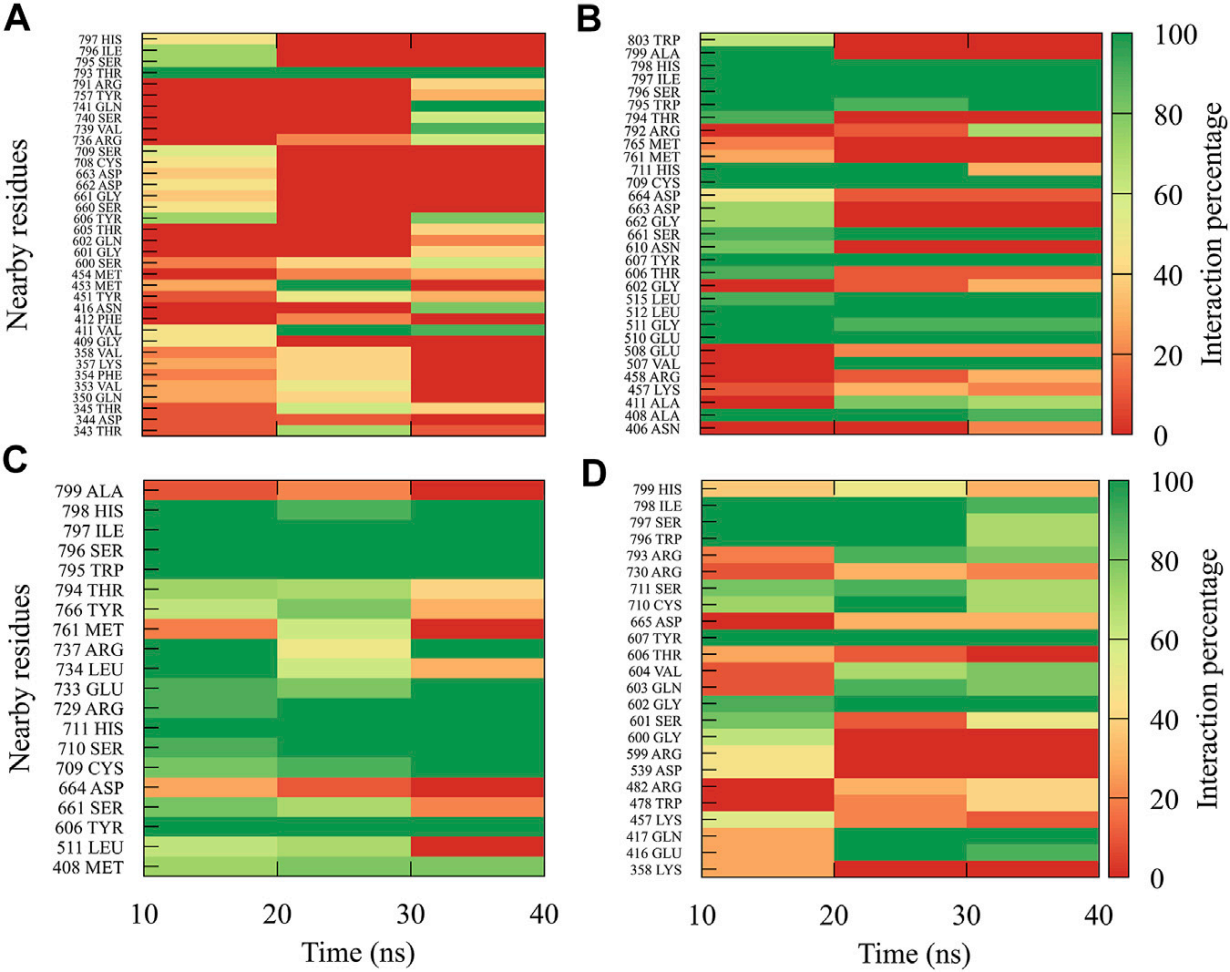
Contact frequency maps for the compound *7sd* **,** bound to the region of the GDD motif in the RdRp domain of the **(A)** DENV1, **(B)** DENV2, **(C)** DENV3, and **(D)** DENV4. Red represents that the contact frequency was 0%, while green represents that the contact frequency was 100%.

For compound *7sd*, bound to the GDD motif region of the RdRp domain, the highest frequencies and numbers of interactions were with DENV3 and DENV2 serotypes (Figures 9B, C), and the lowest frequencies of interactions were for DENV1 and DENV4 serotypes (Figures 9A, D). Also, *7sd* conserved interactions with some amino acids that have been involved in the stabilization of the inhibitor NITD107 crystallized in the GDD motif region and as important for the polymerase activity of the RdRp domain (Noble et al., 2013; Klema et al., 2016), such as Val411, Phe412, Val603, Thr605, Tyr606, Asp663, Asp664, Trp795, and Ile797. To indicate the position of the amino acids involved, the sequence of NS5 DENV3 was taken as reference, according to the alignment (Supplementary Figure S1).

**FIGURE 10:**
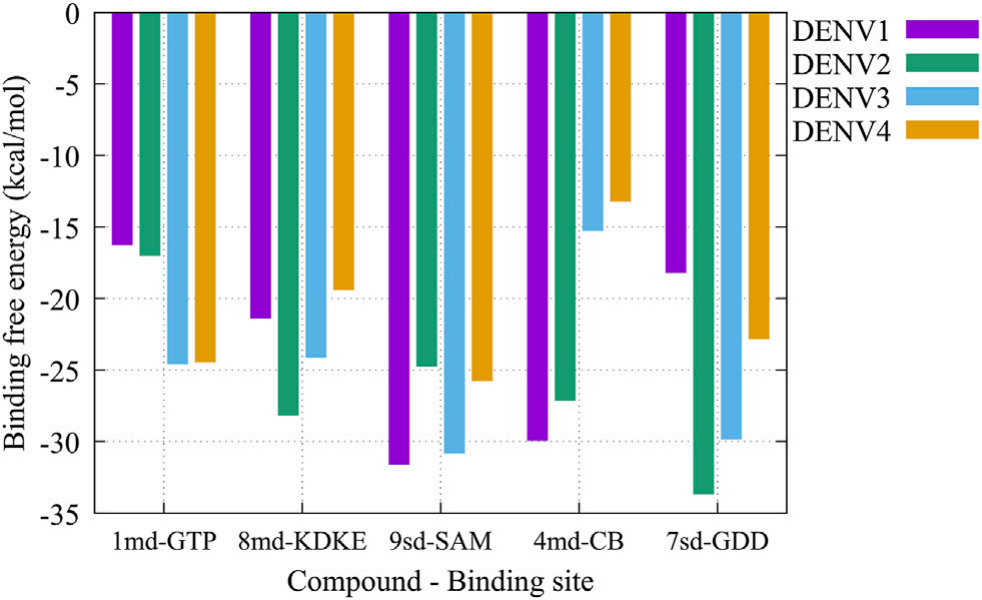
Binding-free energy obtained from the MMPBSA method for the simulated protein–ligand complexes. Major pharmacophore patterns for each binding site.

On the other hand, to analyze possible preferences of the compounds for any of the serotypes, we calculated the binding-free energy from molecular dynamics simulations (Figure 10). Our results suggest that compound *1md* prefers the DENV3 and DENV4 serotypes over the other two serotypes, with DENV3 and DENV4 being the serotypes with the highest ΔG_bind_ values. For compound *8md*, the two best ΔG_bind_ values were for the DENV2 and DENV3 serotypes. For compound *9sd*, the best ΔG_bind_ values were for DENV1 and DENV3 serotypes. For compound *4md*, the best ΔG_bind_ values were for DENV1 and DENV2. Finally, for compound *7sd*, the best values were for the DENV2 and DENV3 serotypes. Thus, in general, we can conclude that the serotypes with the best ΔG_bind_ values were the DENV2 and DENV3 serotypes.

**FIGURE 11:**
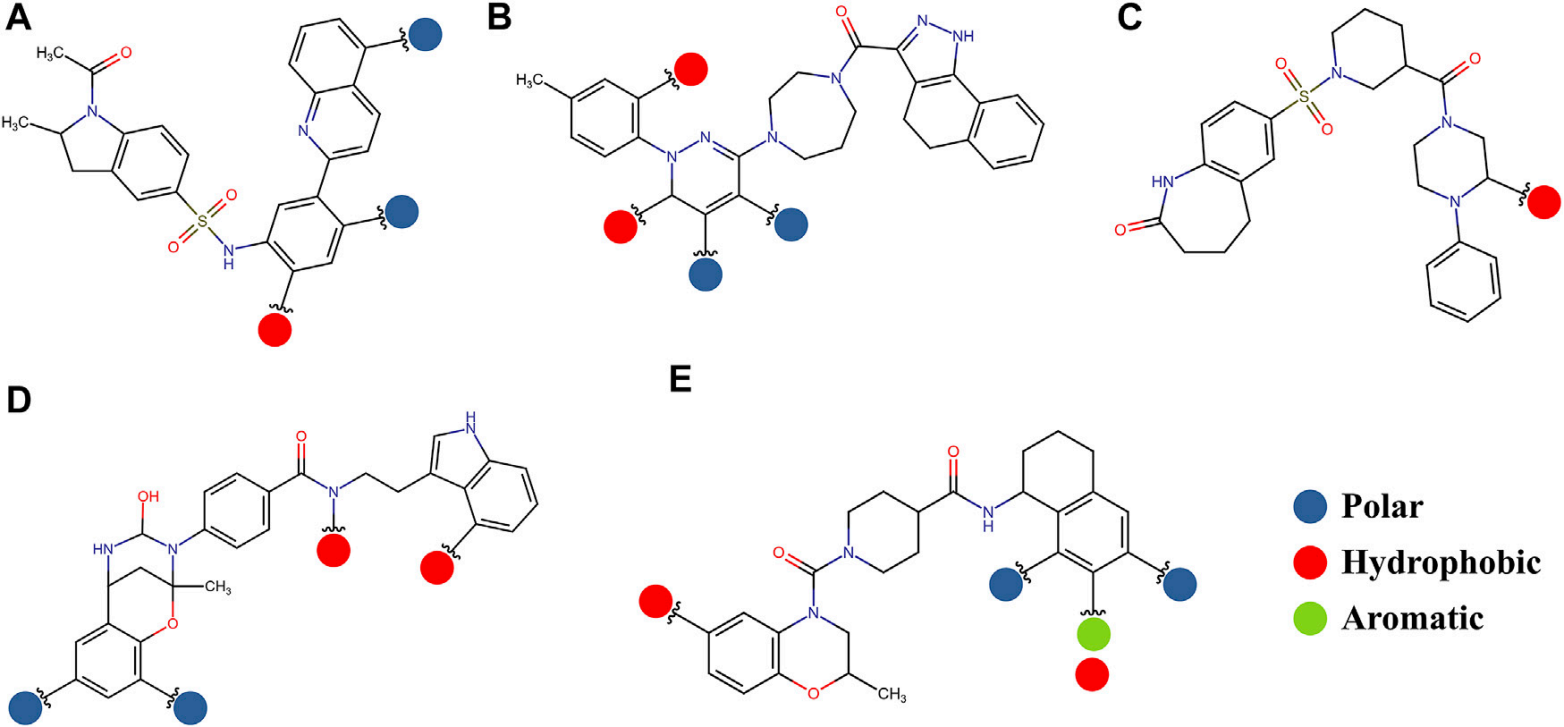
Possible structural modifications for the compound *1md* bound to the GTP-binding site **(A)**, compound *8md* bound to the region of the KDKE tetrad **(B)**, compound *9sd* bound to the SAM-binding site **(C)**, compound *4md* bound to the cavity B **(D)**, and compound *7sd* bound to the region of the GDD motif **(E)**. The polar, hydrophobic, and aromatic patterns are shown as blue, red, and green spheres, respectively. These spheres show the position on each compound where they can be located.

The pharmacophore approximation models were presented as an additional result after selecting the best compounds for each region. The idea behind this analysis was to take advantage of the predicted poses of the best compounds and obtain a pattern of chemical characteristics (Supplementary Figure S3) that can suggest structural modifications of the ligands that can be used to design new molecules in future research (Figure 11).

The prediction of the pharmacophore patterns in the GTP-binding site was predicted using compounds *1md*, *2md*, *5md*, and *1sd* (Table 2), among which *1md* (ID ZINC15827835) being the compound with the best affinity value. In general, four pharmacophore patterns are observed (Supplementary Figure S3A) to consider in compounds with affinity for the GTP-binding site: (I) a highly hydrophobic region, (II) a highly polar region, (III) an aromatic ring with polar and hydrophobic radicals, and (IV) an aromatic region that could be represented by a two-ring fragment, where both can have polar atoms. Based on this, we postulate 3 sites in *1md*, in which 2 polar-type and 1 hydrophobic modifications can be explored (Figure 11A).

The best compounds for the KDKE tetrad are shown in Table 3, where *8md* (ID ZINC15730188) being the compound with the best affinity results. The pharmacophore patterns at this binding site were predicted using compounds *6md*, *8md*, *5sd*, and *6sd* (compounds with the best affinity values). We highlight four pharmacophore patterns for compounds with possible binding in the region of the KDKE tetrad (Supplementary Figure S3B): 1) an aromatic fragment with hydrophobic substituents, 2) an aromatic region with hydrophobic and polar radicals, 3) a highly polar fragment, 4) and a voluminous fragment that can be described by a three-ring fragment, an aromatic one with heteroatoms, followed by a hydrophobic one, and finally a fully aromatic one. Based on this, we postulate four sites in *8md*, in which 2 polar-type and 2 hydrophobic modifications can be explored (Figure 11B).

**TABLE 3:** Best compounds obtained for the KDKE tetrad site of the four DENV serotypes. The best compound is highlighted in bold.

Compound	Molecular structure	Docking score (kcal/mol)			
		DENV1	DENV2	DENV3	DENV4
6md ZINC33106115	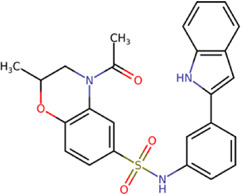	−9.7	−11.1	−9.9	−11.2
7md ZINC15869753	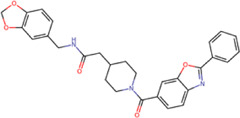	−9.8	−11.0	−9.8	−11.0
**8md ZINC15730188**	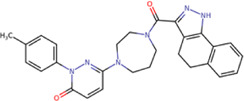	**−10.4**	**−11.5**	**−10.8**	**−11.7**
5sd ZINC21152560	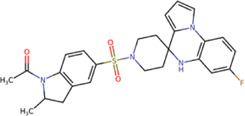	−10.0	−11.1	−10.2	−11.0
6sd ZINC12307816	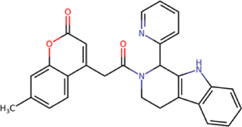	−9.8	−11.0	−10.0	−11.1

Among our results, only two compounds, *9sd* (ID ZINC20943220) and *10sd* (ID ZINC20943169), were in the SAM-binding site (Table 4), both presented affinities of similar values, although *9sd* has the best results. The both compounds were used for pharmacophore prediction because *9sd* and *10sd* have very similar chemical structures and the pharmacophore pattern mostly matches for both compounds. Region I, which is described by the identical part between both molecules, is conserved. Region II suggests that a more elongated aromatic group would be more representative, coinciding with the fact that it is a phenylpiperazine fragment for *9sd* and it is an indole ring for *10sd*, which is a smaller fragment (Supplementary Figure S3C). Based on this, we postulate only one site in *9sd*, in which hydrophobic-type modifications can be explored (Figure 11C).

**TABLE 4:** Best compounds obtained for the SAM-binding site of the four dengue serotypes. The best compound is highlighted in bold.

Compound	Molecular structure	Docking score (kcal/mol)			
		DENV1	DENV2	DENV3	DENV4
**9sd ZINC20943220**	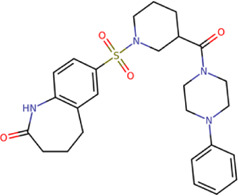	**−9.7**	**−11.3**	**−9.7**	**−11.2**
10sd ZINC20943169	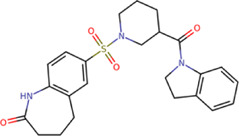	−9.7	−11.1	−9.6	−11.1
SAH^a^	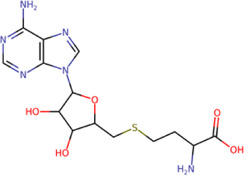	−7.8	−7.9	−8.0	−7.9

a Crystal control: natural subtract of the MTase domain crystallized with the PDB accession code: 5JJR (Lim et al., 2016).

The best compounds for cavity B are shown in Table 5, where *4md* (ID ZINC9835726) being the compound with the best binding-free energy score. Then, in order to describe the chemical environment for inhibitors with binding in cavity B, we calculated a possible pharmacophore using compounds *2md*, *4md*, *3sd*, and *4sd*. In general, five chemical patterns can be described to consider in the structure of the future ligand with binding in cavity B: 1) an aromatic region with polar substituents, 2) a polar region with small hydrophobic groups, 3) an aromatic fragment as a linker, 4) a highly polar region with a hydrophobic character, and 5) a voluminous hydrophobic region (Supplementary Figure S3D). Based on this, we postulate four sites in *4md*, in which 2 polar-type and 2 hydrophobic modifications can be explored (Figure 11D).

**TABLE 5:** Best compounds obtained for cavity B of the four dengue serotypes. The best compound is highlighted in bold.

Compound	Molecular structure	Docking score (kcal/mol)			
		DENV1	DENV2	DENV3	DENV4
1md ZINC15827835	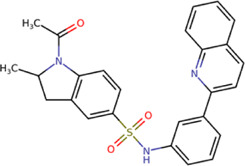	−8.9	−8.8	−8.8	−9.0
2md ZINC15827831	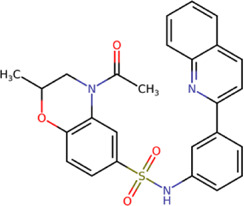	−9.1	−9.0	−9.0	−9.0
3md ZINC15730200	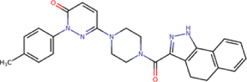	−8.6	−8.8	−8.9	−8.5
**4md ZINC9835726**	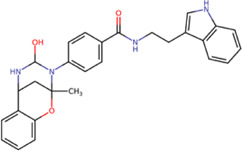	**−9.6**	**−9.5**	**−9.5**	**−9.2**
5md ZINC9268549	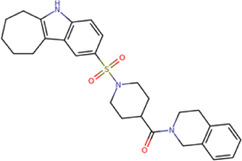	−8.6	−8.7	−8.7	−8.8
6md ZINC33106115	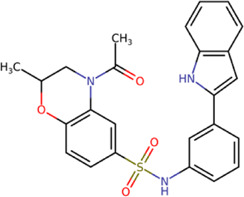	−8.8	−8.8	−8.8	−8.7
7md ZINC15869753	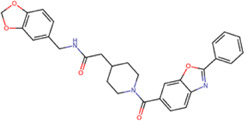	−8.8	−8.4	−8.5	−8.6
3sd ZINC12705528	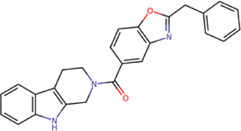	−9.0	−9.1	−9.0	−9.1
4sd ZINC09268580	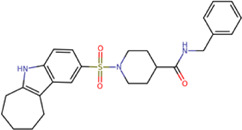	−9.4	−9.0	−9.1	−8.9
PBTZ1^a^	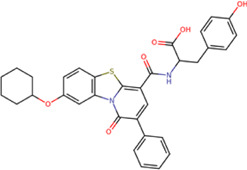	−7.9	−7.6	−7.5	−7.6

a Crystal control: compound with reported anti-dengue activity and with possible binding mode in cavity B (Cannalire et al., 2020).

For the case of the GDD motif site, only 3 compounds were obtained (Table 6), of which *7ds* was the one that presented the best affinity scores. Therefore, the 3 compounds, *8md*, *7sd*, and *8sd*, were used for the prediction of pharmacophores. For this case, three regions are described: 1) a bulky region, which can be described by fragments of two rings, one aromatic ring with heteroatoms, and the other with one hydrophobic ring; 2) a region with a hydrophobic ring and an aromatic ring with substituents polar, thus forming a fragment of two rings; and finally 3) a region that suggests that aromatic-type modifications with hydrophobic substituents can be considered on the aromatic ring of region II (Supplementary Figure S3E). Based on this, we postulate four sites in *7sd*, in which 2 modifications of the polar type and 1 modification of the hydrophobic type were explored, and for this case we found that an aromatic fragment with hydrophobic substitutions could be included, for example, a benzene ring with hydrophobic groups in the para position (Figure 11E).

**TABLE 6:** Best compounds obtained for the GDD motif of the four dengue serotypes. The best compound is highlighted in bold.

Compound	Molecular structure	Docking score (kcal/mol)			
		DENV1	DENV2	DENV3	DENV4
8md ZINC15730188	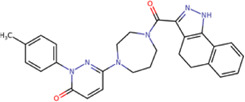	−9.9	−10.4	−10.3	−9.8
**7sd ZINC09405884**	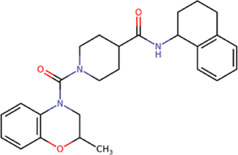	**−10.3**	**−10.6**	**−9.8**	**−10.2**
8sd ZINC21887378	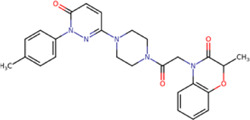	−10.1	−10.2	−9.7	−10.4
NITD107^a^	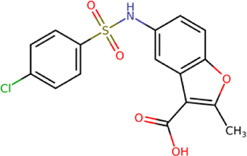	−8,4	−9.2	−8.0	−8.5

a Crystal control: inhibitor of the RdRp domain of DENV3 crystallized with the PDB accession code: 3VWS (Noble et al., 2013).

### Cytotoxicity Assay in Huh-7 Cells

**FIGURE 12:**
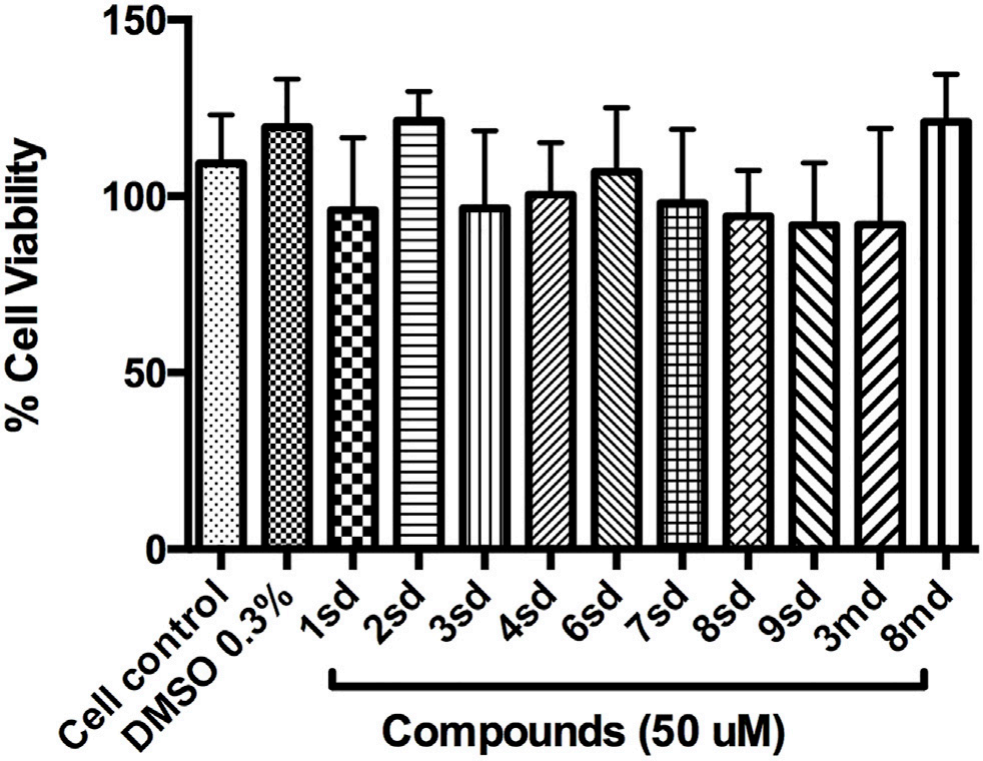
Graph of cell viability of the compounds to 50 µM. Cell control corresponds to cells without treatment, while DMSO 0.3% corresponds to the diluent of the compounds.

Initially, the cytotoxic effect of 10 of the 18 candidate compounds identified in this study was evaluated on the Huh-7 cell line. Cell viability was close to 100% in the presence of the evaluated compounds (Figure 12), without statistically significant differences with respect to the control, indicating that they present CC_50_ greater than 50 µM in Huh-7 cells.

## Discussion

In recent years, many efforts have been made to search for effective antiviral compounds as a treatment against DENV. Among the identified compounds, only a few have been further evaluated in preclinical or clinical trials (Troost and Smit, 2020). The difficulty that has arisen in finding effective treatments is related to the toxicity of some compounds evaluated, as has been reported in other investigations (Lim et al., 2013b; Tay et al., 2013; Caillet-Saguy et al., 2014; Fusco and Chung, 2014; Lim et al., 2015; García et al., 2017), which is why it is important to continue searching for drugs. Computational tools have a large impact in drug discovery because of its fast and promising results (Halim et al., 2017). In the discovery, design, and development of new compounds with putative biological activity, it is common to find research papers that begin with listings or libraries of hundreds of thousands of compounds, which are subsequently reduced to lists of just a few compounds; for this, methodologies based on virtual screening have been proved to be very useful (Jenwitheesuk et al., 2008; Westermaier et al., 2015; Seyedi et al., 2016).

Target identification and validation is the first key stage in the drug-discovery pipeline (Li et al., 2008). In the present study, the DENV NS5 protein was selected as a target. NS5 represents a promising antiviral target (Tripathi and Shrivastava, 2018) to design specific inhibitors with low toxicity (El Sahili and Lescar, 2017). We obtained the three-dimensional structures for all dengue virus serotypes, based on PDB accession code: 5JJR. The identity of sequences between NS5 was consistent with that of the sequence identities reported in other studies (García et al., 2017; Sampath and Padmanabhan, 2009). This crystal structure was an adequate template for the first stage of this study, finding values close to 1 in the measurement of the quality of each model according to GMQE and QMEANDisCo and valid stereochemical quality, confirmed with the Ramachandran plot. The structural alignment showed small RMSD values, being the lowest between DENV1–DENV4 and the biggest between DENV2–DENV3. The difference between these global topologies is related to the differences about the sequence (Saw et al., 2015). It has been shown that NS5 adopts multiple conformations owing to its flexible linker and that DENV4 NS5 is more compact and less flexible compared with NS5 from DENV1 to DENV3 (Saw et al., 2015; Subramanian et al., 2016). A ten-residue linker in the NS5 protein is important in communication between the MTase and RdRp domain (Zhao et al., 2015b; Saw et al., 2015). According to the alignment in this region, the higher sequence identity was 60% between NS5 linker DENV1–DENV3 and less between NS5 linker DENV1–DENV2, DENV1–DENV4, and DENV2–DENV4, with 30%, indicating variation in different amino acid in all serotypes, found only conserved E267 and E269 according to sequence of DENV3 (Supplementary Figure S1). Together with the high percentages of identity between sequences and the high conservation of the global topology of the protein, it is possible to think of drugs that can act on the NS5 protein in the four DENV serotypes. The Z-score values for each NS5 structure (Figure 1B) indicate that the built structures are comparable to other proteins resolved by X-Ray and NMR, indicating an acceptable overall quality, because *Z*-scores outside a range characteristic for native proteins indicate erroneous structures (Wiederstein and Sippl, 2007). Also, it is observed that the most distant model, with respect to NS5 DENV3, is DENV2; however, all the models obtained included most of the amino acids of NS5, allowing the models to be reliable and comparable. Additionally, the Ramachandran plot results for each NS5 allowed to continue with the virtual screening, suggesting that the structures of each model displayed a valid structural quality, according to torsion angles (Hollingsworth and Karplus, 2010).

After molecular docking in the DrugDiscovery@TACC web portal, all compounds were analyzed through physicochemical predictions and ADMET. One of the most important/common parameters in drug development is the Lipinski’s rule, which plays an important role because it reveals that if the selected compounds possess the properties of possible drugs and they may be used in the future as drug candidates (Ahmad et al., 2020). As with the development of drug discovery, it was realized that it is important to filter and optimize the ADMET properties for drugs at an early stage, which has been accepted and widely used to reduce the attrition rate in drug research and development (Wu, F et al., 2020). So, starting with a total of 22 compounds with possible multi-domain binding and 499 compounds with possible single-domain binding in the NS5 protein of DENV, we came up with a list of 18 compounds with varied chemical structure (Tables 2–6). For the binding sites in which we have controls (GTP-binding site, SAM-binding site, cavity B, and GDD motif), the molecular docking results suggest that all our compounds have an affinity for the binding site greater than their respective control. In the case of GTP- and SAM-binding sites, the results suggest a competitive-type interaction, presenting much higher affinities than natural substrates. For the case of cavity B, we used one compound reported by Cannalire et al., called PBTZ1. It presented a mean effective concentration (EC_50_) to inhibit DENV2 replication of 2.1 ± 0.22 μM (Cannalire et al., 2020). For the binding site of the GDD motif, we use the crystallized compound by Noble et al., in the RdRp domain of DENV3 (PDB accession code: 3VWS) called NITD107 as control. It inhibits the RdRp activity of DENV4 with an IC_50_ value of 113 μM and inhibits DENV2 replication with an IC_50_ value of 100 μM (Noble et al., 2013). Thus, in general, our molecular docking results suggest that all our compounds docked on cavity B and GDD motif region would have a better affinity than the PBTZ1 and NITD107 compounds, which in turn suggests that they, in theory, could have a better *in vitro* effect.

Subsequently, making use of the best compound for each binding site, we carried out a new calculation of the binding-free energy but this time from molecular dynamics simulations. First, our simulations show that, generally speaking, the backbone of the protein ranges roughly between RMSD values of 0.2–0.4 nm. However, for some time periods and specific serotypes, RMSD values of ∼0.5 nm were reached. For example, for compound *7sd* in serotype DENV4. Here, it is necessary to mention that since the simulation time was only 40 ns, new simulations with a much more extensive sampling are necessary to delve into the behavior of each of the proteins once they interact with these compounds, although it has been reported that even shorter simulation times may be suitable for performing junction-free energy calculations using MMPBSA (Xu et al., 2013; Genheden and Ryde, 2015; Wang et al., 2019). In addition, other additional analysis could be useful to deepen the selectivity of these compounds (Al-Sha’er and Taha, 2010; Al-Sha’er and Taha, 2021). Recently Wu, J et al., reported the crystallographic structure of the DENV2 NS5 protein in two distant conformations (Wu, J et al., 2020): the structure with PDB accession code: 6KR2 adopts a conformation similar to the homologous protein in the Zika virus, and the structure with PDB accession code: 6KR3 adopts the same conformation as the crystallized structure for serotype DENV3 with PDB accession code: 5JJR. The RMSD value between the 6KR2 and 6KR3 crystals is ∼0.57 nm, while the RMSD value between the 6KR2 and 5JJR crystals is ∼0.91 nm (values calculated with the Chimera program). Zhao et al., performed molecular dynamics simulations to study the role of linker amino acid mutations in the DENV3 NS5 protein and obtained an RMSD for the wild-type protein that converges around 0.5 nm after 20 ns (Zhao et al., 2015b). So, if we consider that the DENV NS5 protein can modify its structure reaching RMSD values lower than 1 nm, it is feasible to think that our RMSD values could be considered natural fluctuations of the protein. In agreements, from the Rg plots (Supplementary Figure S4), it could be observed that the compaction or stiffness of all the serotypes with all the compounds oscillates in a range of 3–3.2 nm, being the complexes of serotype 3 those that presented minor changes in their values of the Rg in time with respect to the other serotypes.

The best compounds conserved interactions with important amino acids within each of the studied binding sites (Figures 5–9). The DENV2 and DENV3 serotypes were the ones with the highest frequencies and the highest number of contacts with the compounds. In addition, it can be observed that the DENV4 serotype tends, in a general way, to present interactions with very low frequencies with respect to the other serotypes. From now on, it could be hypothesized which will be the serotypes that present the best free binding energies with the compounds. In this order of ideas, our contact frequency results have a correlation with the results of the ΔG_bind_ calculation (Figure 10), since the DENV4 serotype presented the lowest frequency of interactions and also the lowest values were obtained for ΔG_bind_. In this way, more economical computational analyses such as the frequency of interactions from classical molecular dynamics simulations could be used to filter compounds in this type of methodologies. Also, using the best compounds and analyzing their pharmacophore patterns, we postulate specific sites on the structure of the best molecule of each binding site (Figure 11) to be able to carry out modifications that can lead to increase the affinity for its binding site in the DENV NS5 protein.

According to our results, we found compounds with the possibility of binding to the two enzymatic domains of NS5 protein, which is interesting considering that both functions (MTase and RdRp) have been investigated as antiviral targets. Targets with multiple binding sites (prerequisites or allosteric) are of increasing importance in the drug design (Hetényi and Bálint, 2020). The exploration of multiple binding sites is of great importance in pharmacology (Hetényi and Bálint, 2020; Yuan et al., 2020) and has been a strategy considered in various studies (Hammoudeh et al., 2009; Ludlow et al., 2015). Most studies have focused on the function of NS5 as RdRp (Troost and Smit, 2020) because this activity is absent in the host cell (Galiano et al., 2016), and this is promising to design specific inhibitors with low toxicity (El Sahili and Lescar, 2017); and within RdRp, cavity B has been considered a site to be explored for the drug design (Malet et al., 2008; Alhossary et al., 2018); however, the MTase domain is also reported as an attractive strategy in the anti-flavivirus drug design (Santos et al., 2020), and regions such as GTP and SAM pockets are obvious targets for antiviral development since they have both been shown to bind to low-molecular-weight ligands (Lim et al., 2015).

The idea of looking for drugs for all serotypes must consider the differences between them because although any serotype is equally able to cause dengue, serotype differences have been postulated to lead to differences in pathogenesis such as the case for DENV2 which has been related with severe dengue (Trujillo-Correa et al., 2019); however, DENV inhibitors that protect toward two or three serotypes should not be neglected for further testing. Thus, serotype-specific treatment may help to treat serotype confirmed DENV patients, and when more antiviral drugs are available it may be possible to achieve a pan-protective effect *via* drug combinational therapy (Troost and Smit, 2020).

On the other hand, the *in silico* assays have served as a starting point for the identification of potential compounds as inhibitors; many studies report this strategy as a good means for the discovery and development of drugs for different diseases. Some example for this is the use of these strategies to search, from libraries, for natural compounds that inhibit RdRp of DENV (Galiano et al., 2016) and also the identification of phytochemical compounds reported for various flaviviruses, on non-structural proteins of DENV (Tahir ul Qamar and Muner, 2019).

On the other hand, with respect to *in vitro* assays, 10 of the 18 candidate compounds were acquired and subsequently evaluated on Huh-7 cells, in order to determine their effect on cell viability. The Huh-7 cell line is derived from hepatocytes, which represent a target cell during natural DENV infection. These cells have been used in previous studies of dengue–host cell interaction (Pando-Robles et al., 2014). CC_50_ is defined as the concentration that causes a 50% reduction or inhibition of cell viability, or that causes 50% cytotoxicity (Dewi et al., 2019). The results indicated that none of the compounds were cytotoxic at the highest concentration evaluated, which implies a CC_50_ for each of them above 50 μM, during the first 24 h of exposure (Figure 12). The evaluation of all concentrations reflected that cell viability remained above 80% for most of the compounds, which validates the evaluation of these compounds in antiviral tests on this cellular model.

In this study, we propose 18 compounds, identified in *in silico*, as possible candidates for the *in vitro* evaluation of the antiviral effect in the four serotypes of dengue virus. The compounds identified in this work bind to different regions of the DENV NS5 protein, such as cavities that are allosteric sites and active sites in the MTase and RdRp domains. However, these compounds are not nucleotide analogs, yet they interact on important sites of the NS5 protein and may be promising compounds to be evaluated by *in vitro* assays.

### Future Outlook

According to our results, our interest is to evaluate the identified compounds in this study (18 compounds) *in vitro*. It is worth mentioning that all the compounds presented here (18) were selected under a rational identification criterion, for which the classification of “best compounds” according to the *in silico* analyses was supported by the binding-free energies that they presented in the selected regions of the NS5 protein; however, all the identified compounds correspond to compounds with desirable physicochemical and pharmacological properties to have been considered as candidates to be evaluated *in vitro*.

The experimental validation of the identified compounds has been carried out with prior identification of cellular toxicity on Huh-7 cells as an ideal model of dengue infection. Subsequently, it is intended to evaluate the effect on DENV by means of plaque-forming units (PFUs), on the synthesis of viral proteins and RNA and on the activity of the viral NS5 protein.

So far, we have evaluated the cytotoxicity of some of these compounds on Huh-7 cells, as *1sd*, *2sd*, *3sd*, *4sd*, *6sd*, *7sd*, *8sd*, *9sd*, *3md*, and *8md* (compounds that were possible to purchase from the seller). According to preliminary results, the compounds do not generate cytotoxic effect in lower concentrations to 50 μM, finding cell viability greater than 80% for most of these, which validates the evaluation of these compounds in other *in vitro* assays. A preliminary analysis of the effect of these compounds against Huh-7 cells infected with DENV2, showed that for compounds *8md* and *9sd*, there is a statistically significant difference (*p*-value <0.01) between the percentage of plaque-forming units (PFUs) of DENV compared to the control of DMSO 0.3%, indicating that was a reduction by treatment. On the other hand, other compounds identified in this study, such as compounds *6sd* and *3md* reduce Log PFUs, with a behavior similar to that presented by compound NITD-008, a nucleotide analog that it potently inhibits replication in DENV (Yin et al., 2009a; Lim et al., 2013b) and that was used as an experimental control; furthermore, the compound *3md* has been found to reduce the percentage of infected cells and the number of viral copies. The *in vitro* antiviral assays are in process.

## Conclusion

We performed a computational screening of several drug-like compounds with potential effects against the NS5 protein of the dengue virus. We report two virtual screening strategies focused on the search for compounds with binding to multiple sites within the same protein, called multi-domain compounds, and compounds of classical inhibition or those that bind to a single site within the NS5 protein, called single-domain compounds. As inclusion and exclusion criteria, we developed a series of filters that allowed us to recognize possible structural risks in the selected compounds to minimize complications in future experimental trials against DENV. Starting from a list of ∼642,759 commercially available drug-like compounds for each strategy, we found 8 compounds with multi-domain binding and 80 with single-domain binding. Then, we identified the best compound for each region and analyzed their interaction with the four serotypes. According to our results, we highlight a short list of 18 compounds as the most promissory for future research. Additionally, we suggest that contact frequency analysis can be useful when filtering compounds from molecular dynamics simulations, being computationally cheaper than a calculation of free binding energies, and in the evaluation *in vitro* of 10 of these compounds we found that they are not cytotoxic in the Huh-7 cell line below 50 μM. We are aware that experimental validation, more extensive simulations, and robust thermodynamic studies can be useful in order to validate our hypothesis and to expand the search for compounds with antiviral activity.

## Data Availability

The original contributions presented in the study are included in the article/Supplementary Material; further inquiries can be directed to the corresponding author.
