# Supplementary material for: Virtual Screening of Drug-Like Compounds as Potential Inhibitors of the Dengue Virus NS5 Protein
Source: Front Chem. 2022 Feb 10;10:637266. doi: 10.3389/fchem.2022.637266 (PMC8867075; doi:10.3389/fchem.2022.637266)
Supplement: Supplementary file 1 [file DataSheet1.pdf]

Supplementary material for: Virtual screening of drug-like compounds as potential inhibitors of dengue virus NS5 protein

Leidy L. García-Ariza[1*], **Cristian Rocha-Roa**[2,3], **Leonardo Padilla-Sanabria**[1] **and Jhon C. Castaño-Osorio**[1]

[1] Grupo de Inmunología Molecular, Centro de investigaciones Biomédicas, Universidad del Quindío, Armenia, Colombia.

[2] Grupo de Parasitología Molecular, Centro de investigaciones Biomédicas, Universidad del Quindío, Armenia, Colombia.

[3] Biophysics of Tropical Diseases, Max Planck Tandem Group, Universidad de Antioquia, Medellín, Colombia.

*** Correspondence:**
Leidy Lorena García Ariza, llgarcia@uniquindio.edu.co.

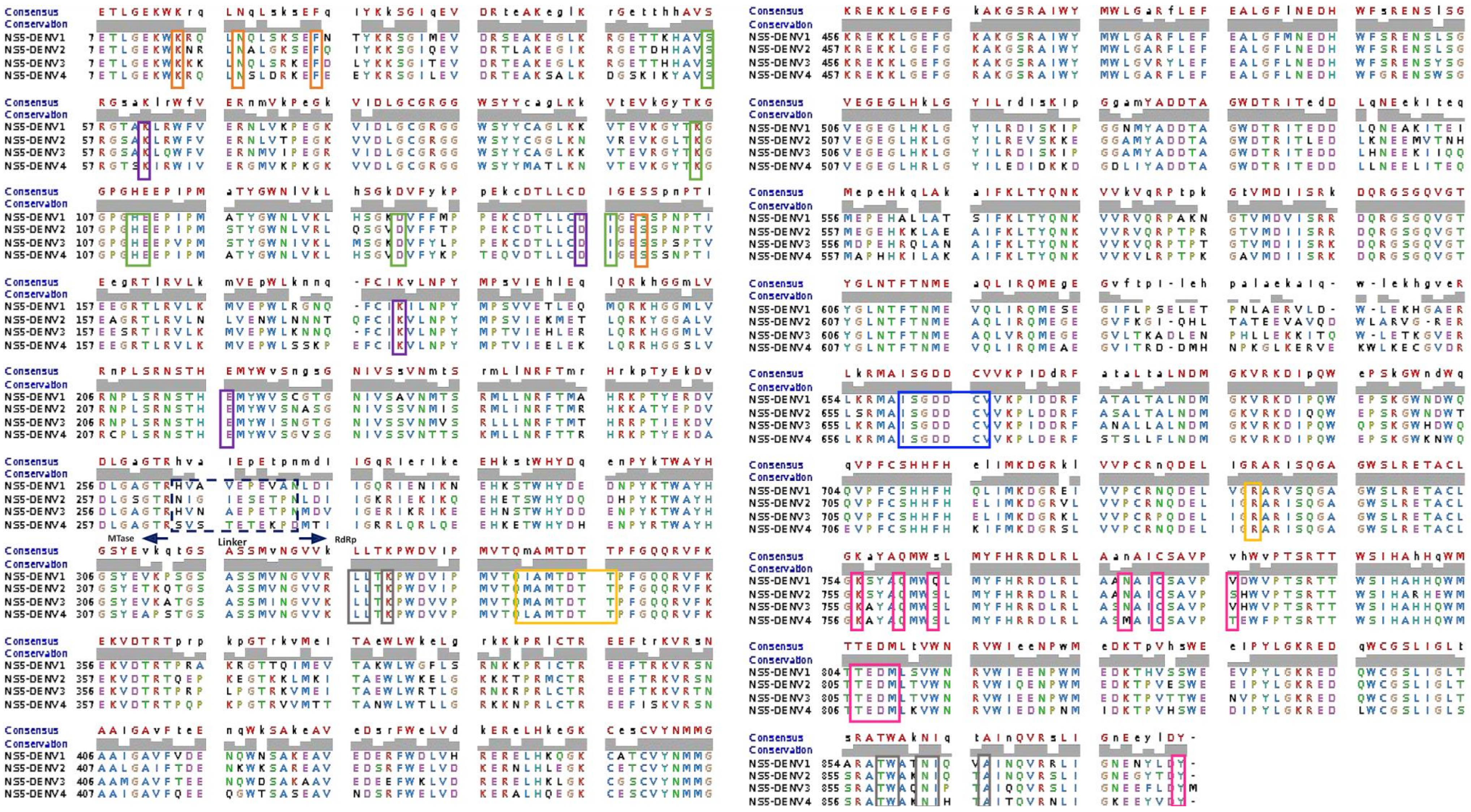
Consensus
Conservation
NS5-DENV1
NS5-DENV2
NS5-DENV3
NS5-DENV4
MTase
Linker
RdRp

Figure S1. Multiple sequence alignment of NS5 protein of dengue virus. The consensus sequences were obtained with Bioedit using the sequences reported in Virus Variation (NCBI) for each serotype (DENV1, 2 and 4). NS5 DENV3 sequence corresponds to PDB accession code: 5JJR in Protein Data Bank. In the boxes are indicated the amino acids included for molecular docking. In orange GTP binding site (K12, N16, F23, S148 (Dong et al., 2008; Lim et al., 2015)), in green SAM binding site (S54, K103, H108, E109, D129, I145 (Dong et al., 2008; Lim et al., 2015)), in purple KDKE tetrad (K59, D144, K179, E215 (Zhou et al., 2007; Dong et al., 2008; Zhao et al., 2015a; Zhao et al., 2015c), in pink Cavity A (K755, Q759, S762, N776, C779, V784, T805, E806, D807, M808, Y881 (Malet et al., 2008 ; Zou et al., 2011)), in gray Cavity B (L325, L326, K328, T857, W858, N861, I862, A865 (Malet et al., 2008; Zou et al., 2011)), in yellow RNA tunnel (M339, A340, M341, T342, D343, T344, T345, R736 (Yap et al., 2007; Niyomrattanakit et al., 2010; Galiano et al., 2016)), and blue GDD motif (I659, S660, G661, D662, D663, C664, V665 (Galiano et al., 2016)). In this alignment, from position E7 to R262 is Mtase domain, and from M273 to M883 is RdRp domain, in NS5-DENV3 (Zhao et al., 2015). In the position H263-N272 (in DENV1 and DENV3) we can find ten residues of the linker region, these are important in communication between MTase and RdRp domain (Zhao et al., 2015b; Saw et al., 2015). *Note: the position of the amino acids in this alignment, for each DENV serotype, differs from those reported in the aforementioned investigations, but they correspond to the same residue. In this alignment, all the sequences were matched to NS5-DENV3 sequence in E7 amino acid in order to identify conserved residues in all serotypes.*

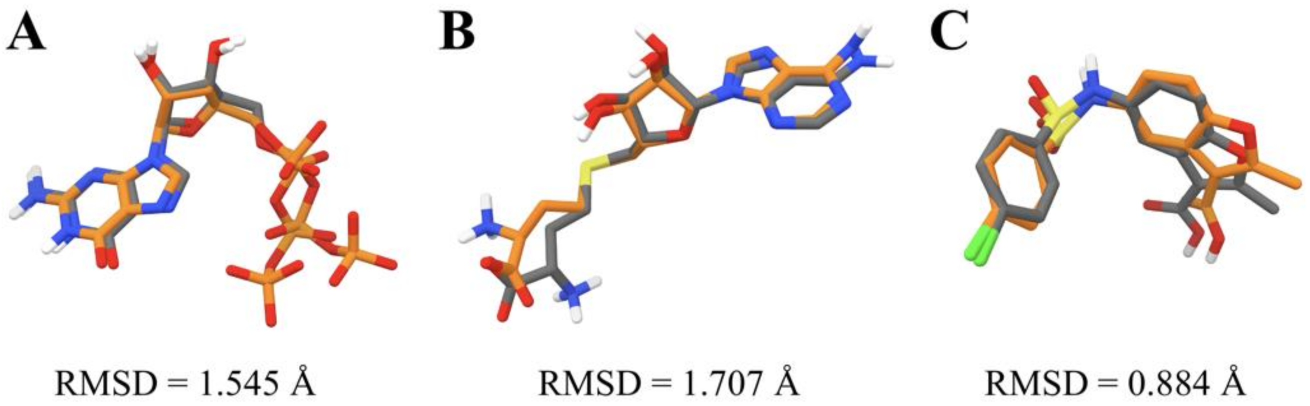

Figure S2. Validation by re-docking of the regions (A) GTP binding site, (B) SAM binding site and (C) GDD motif. The crystallized conformation is shown in gray and the predicted by Autodock Vina in orange, in addition the RMSD value between the experimental pose and the crystallographic in Angstroms is presented.

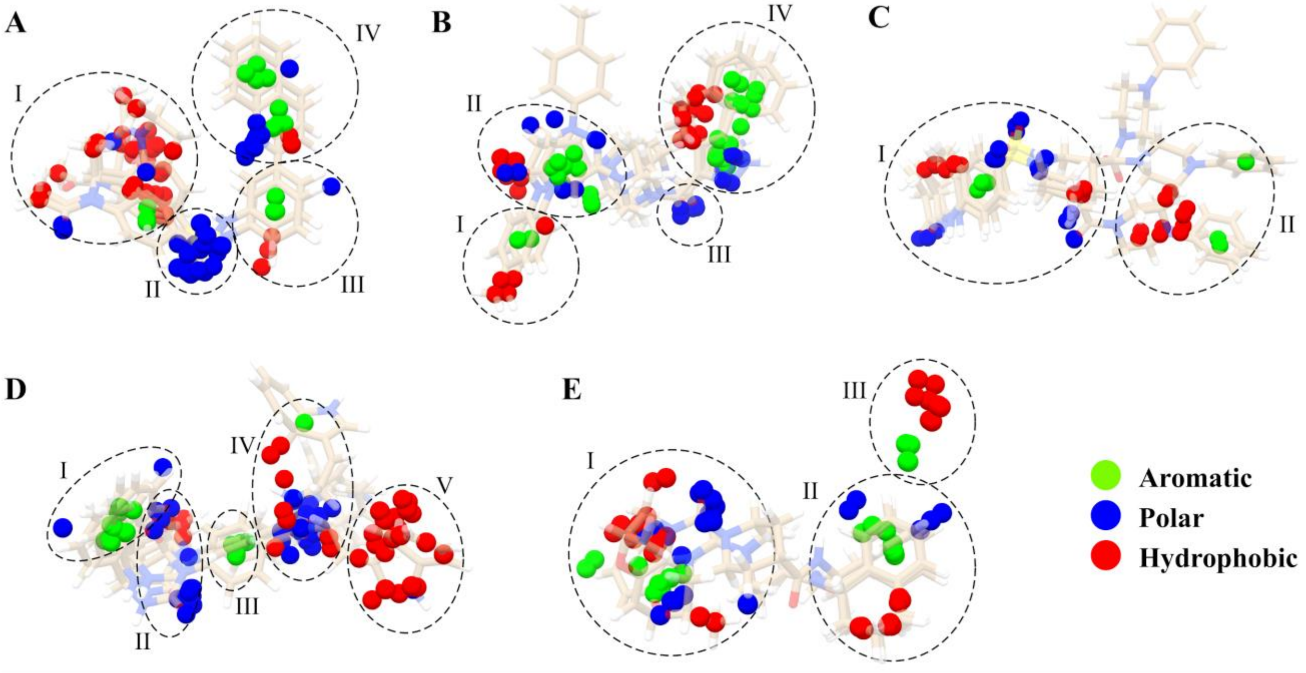

Figure S3. Pharmacophore patterns found in (A) GTP binding site, (B) KDKE tetrad (C) SAM binding site, (D) Cavity B, and (E) GDD motif. The best compound in each region is shown in transparent sticks in its poses obtained for all serotypes. The main patterns at each binding site are circled. The Aromatic, polar and hydrophobic regions are shown as green, blue, and red spheres, respectively.

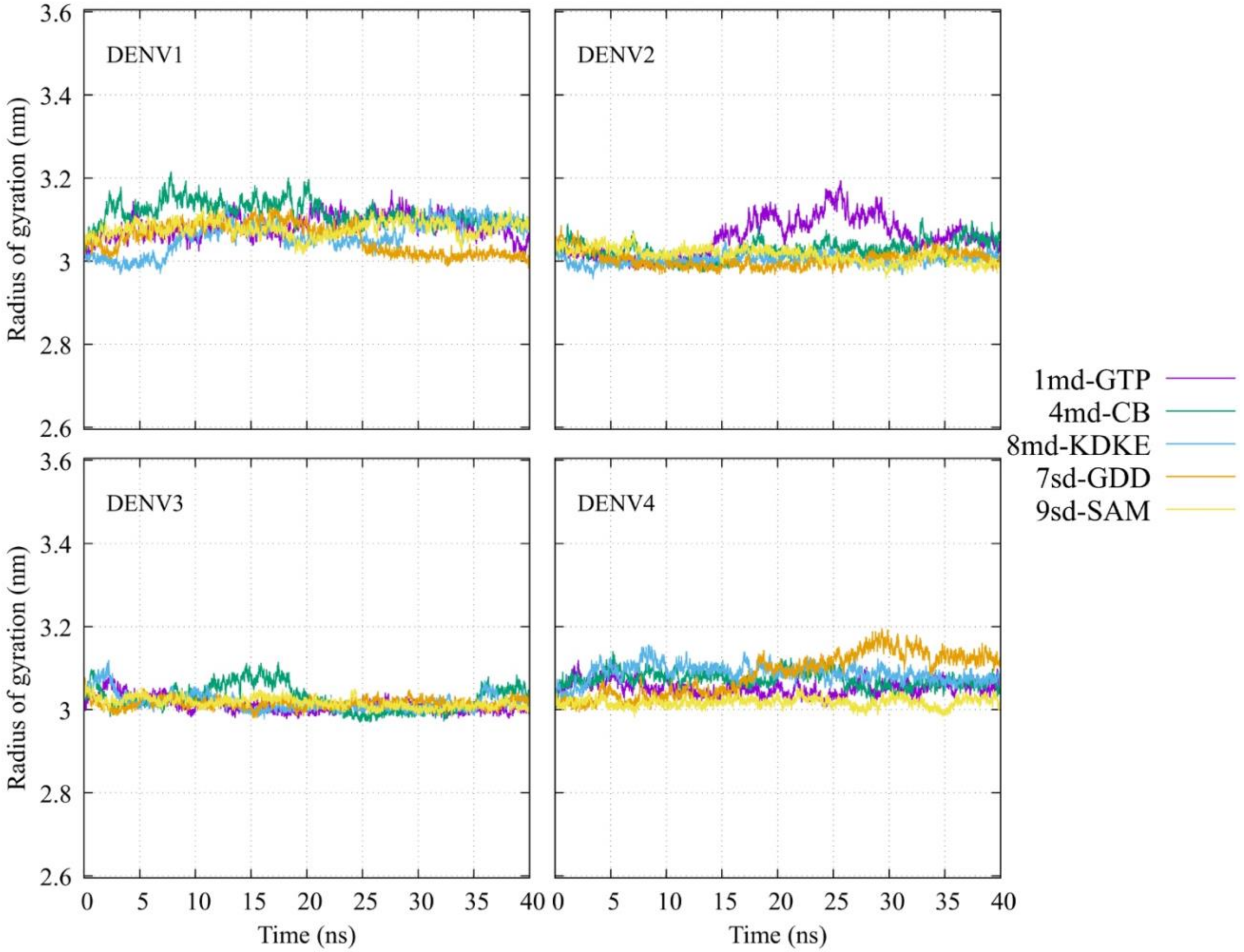

Figure S4. Radius of gyration (Rg) as a function of the time for all the simulated protein-ligand complexes. Decreases in Rg values suggest "compaction", and increases in Rg values suggest an "expansion" of the protein.

References

Dong, H., Zhang, B., and Shi, P.Y. (2008). Flavivirus methyltransferase: a novel antiviral target. *Antiviral Res*. 80,1-10. doi: 10.1016/j.antiviral.2008.05.003.

Galiano, V., Garcia-Valtanen, P., Micol, V., and Encinar, J.A. (2016). Looking for inhibitors of the dengue virus NS5 RNA-dependent RNA-polymerase using a molecular docking approach. *Drug Des Devel Ther*. 10,3163-3181. doi: 10.2147/DDDT.S117369.

Lim, S.P, Noble, C.G., and Shi, P.Y. (2015). The dengue virus NS5 protein as a target for drug discovery. *Antiviral Res*. 119,57-67. doi: 10.1016/j.antiviral.2015.04.010.

Malet, H., Massé, N., Selisko, B., Romette, J.L., Alvarez, K., Guillemot, J.C., et al. (2008). The flavivirus polymerase as a target for drug discovery. *Antiviral Res*. 80,23-35. doi: 10.1016/j.antiviral.2008.06.007.

Niyomrattanakit, P., Chen, Y.L., Dong, H., Yin, Z., Qing, M., Glickman, J.F., et al. (2010). Inhibition of dengue virus polymerase by blocking of the RNA tunnel. *J Virol*. 84,5678-86. doi: 10.1128/JVI.02451-09.

Saw, W.G., Tria, G., Grüber, A., Subramanian Manimekalai, M.S., Zhao, Y., Chandramohan, A., et al. (2015). Structural insight and flexible features of NS5 proteins from all four serotypes of Dengue virus in solution. *Acta Crystallographica Section D Biological Crystallography*. 71, 2309–2327. doi:10.1107/s1399004715017721.

Yap, T.L., Xu, T., Chen, Y.L., Malet, H., Egloff, M.P., Canard, B., et al. (2007). Crystal structure of the dengue virus RNA-dependent RNA polymerase catalytic domain at 1.85-angstrom resolution. *J Virol*. 81, 4753-65. doi: 10.1128/JVI.02283-06.

Zhao, Y., Soh, T.S., Zheng, J., Chan, K.W., Phoo, W.W, Lee, C.C., et al. (2015a). A crystal structure of the Dengue virus NS5 protein reveals a novel inter-domain interface essential for protein flexibility and virus replication. *PLoS Pathog*.11:e1004682. doi: 10.1371/journal.ppat.1004682.

Zhao, Y., Soh, T.S., Chan, K.W., Fung, S.S., Swaminathan, K., Lim, S.P., et al. (2015b). Flexibility of NS5 Methyltransferase-Polymerase Linker Region Is Essential for Dengue Virus Replication. *J Virol*. 89,10717-21. doi: 10.1128/JVI.01239-15.

Zhao, Y., Soh, T.S., Lim, S.P., Chung, K.Y., Swaminathan, K., Vasudevan, S.G., et al. (2015c). Molecular basis for specific viral RNA recognition and 2'-O-ribose methylation by the dengue virus nonstructural protein 5 (NS5). *Proc Natl Acad Sci U S A*.112:14834-9. doi: 10.1073/pnas.1514978112.

Zhou, Y., Ray, D., Zhao, Y., Dong, H., Ren, S., Li, Z., et al. (2007). Structure and function of flavivirus NS5 methyltransferase. *J Virol*. 81,3891-903. doi: 10.1128/JVI.02704-06.

Zou, G., Chen, Y.L., Dong, H., Lim, C.C., Yap, L.J., Yau, Y.H., et al. (2011). Functional analysis of two cavities in flavivirus NS5 polymerase. *J Biol Chem*.286,14362-72. doi: 10.1074/jbc.M110.214189.
